# Toward a digital urinary biopsy for early risk stratification in diabetic kidney disease: Anchoring shed cells, vesicles, and cell‐free DNA to spatial kidney cell states

**DOI:** 10.1002/ctm2.70866

**Published:** 2026-10-28

**Authors:** Juan Wu, Ping Sheng, Ying Lu, Ke Chen

**Affiliations:** ^1^ Department of Geriatrics Xiangya Hospital Central South University Changsha Hunan China; ^2^ National Clinical Research Center for Geriatric Disorders Xiangya Hospital Central South University Changsha Hunan China; ^3^ Department of General Medicine Xiangya Hospital Central South University Changsha Hunan China

**Keywords:** cell‐free DNA methylation, diabetic kidney disease, extracellular vesicles, single‐cell transcriptomics, urinary liquid biopsy

## Abstract

**Background:**

Diabetic kidney disease (DKD) is the leading cause of end‐stage kidney disease worldwide. However, albuminuria and estimated glomerular filtration rate (eGFR), which define and stage DKD, report established injury and do not resolve the cellular heterogeneity underlying variable progression. Urine, the only biofluid to traverse every nephron segment, carries shed cells, extracellular vesicles (EVs) and cell‐free DNA (cfDNA), each retaining information about its cell of origin.

**Main body:**

This review develops the concept of a digital urinary biopsy, in which the three compartments are read together against spatial single‐cell kidney atlases to infer non‐invasively which cell states are active and whether niche‐associated programs are engaged, an approach we term atlas‐anchored molecular profiling. For each compartment, we tabulate study population, sample size, processing, assay, performance and validation status. Because shed‐cell RNA, selectively sorted vesicle cargo and cfDNA methylation differ in origin, measurement scale and bias, each requires its own reference matrix, sampling function and background terms. All three carry renal information in feasibility studies, yet none is a validated clinical assay in DKD. Moreover, a cfDNA methylation reference at the required resolution is lacking, and no named computational method has been tested on urine.

**Conclusion:**

We therefore present the framework as a structured research hypothesis and define the required validation, from analytical and clinical validity to prospective demonstration of utility, with non‐albuminuric DKD, which albuminuria‐based screening misses, as the index use case. It offers a testable route to cell‐resolved, non‐invasive staging of DKD rather than a demonstrated capability.

## Key points

- Urine carries shed cells, EVs and cfDNA from the whole nephron.
- Anchoring all three to spatial kidney atlases could infer active cell states non‐invasively.
- Each compartment needs its own reference, sampling function and background terms.
- Validation, cost and a compact deployable panel remain unresolved.

## Graphical abstract

In diabetic kidney disease, one urine specimen carries shed cells, extracellular vesicles and cell‐free DNA released along the whole nephron. Reading all three against spatial kidney atlases is proposed as a testable route to cell‐resolved, non‐invasive risk stratification, and the clinical outputs shown are not yet demonstrated.

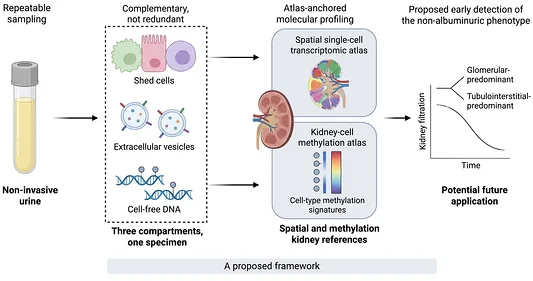

## 1 INTRODUCTION

Diabetic kidney disease (DKD), historically termed diabetic nephropathy, is the leading cause of chronic kidney disease (CKD) and of treated kidney failure worldwide, accounting for roughly half of all incident ESKD and developing in approximately 40% of people with type 2 diabetes (T2D) and 30% with type 1 diabetes (T1D).^1^, ^2^, ^3^ Its burden tracks the global rise in diabetes, and Global Burden of Disease analyses document rising prevalence, disability‐adjusted life years, and mortality, with widening between‐country inequality.^2^, ^4^ Despite advances that include renin–angiotensin system (RAS) blockade, sodium‐glucose cotransporter‐2 (SGLT2) inhibition, shown by Heerspink et al.^5^ to slow progression across the CKD spectrum, and nonsteroidal mineralocorticoid receptor antagonism, shown by Bakris et al.^6^ to reduce kidney outcomes in T2D, many patients still progress to kidney failure, largely because intervention begins only after irreversible structural injury has accrued.^7^, ^8^, ^9^, ^10^ This late start reflects the limitations of the biomarkers that define and stage the disease. For decades, the Mogensen paradigm framed diabetic nephropathy as a stereotyped sequence from glomerular hyperfiltration through microalbuminuria and proteinuria to declining filtration.^11^ Albuminuria and the eGFR, arranged in the Kidney Disease Improving Global Outcomes (KDIGO) risk matrix, remain the stratification basis.^8^ Both, however, are late and nonspecific: albuminuria reflects glomerular barrier disruption already established, and creatinine‐based eGFR is insensitive to early parenchymal loss because of renal functional reserve.^8^, ^10^ The classical sequence has therefore been challenged. Afkarian et al.^12^ showed that mortality risk in diabetes rises with kidney disease independently of albuminuria, and population studies, beginning with Kramer et al.,^13^ established the non‐albuminuric phenotype as common rather than exceptional.^14^ This non‐albuminuric phenotype, defined by eGFR below 60 mL/min/1.73 m^2^ with normal albumin excretion, accounts for a sizeable proportion of DKD; a meta‐analysis by Yang et al.^15^ placed its prevalence at 20% to 40%, varying by population and definition. This is a functional definition and not an aetiological one. Reduced filtration with normal albumin excretion may equally reflect vascular disease, ageing, medication effects, or a prior episode of acute kidney injury, and separating diabetic from non‐DKD on clinical grounds is imperfect: across 26 biopsy studies comprising 2012 patients with T2D, retinopathy identified diabetic nephropathy with a pooled sensitivity of 0.65 and specificity of 0.75, proliferative retinopathy reaching a specificity of 0.98 but a sensitivity of only 0.25.^16^ Study of this phenotype therefore requires repeated measurement of albumin excretion and filtration rather than single values, since microalbuminuria regressed in 58% of patients over six years in one prospective cohort,^17^ together with diabetes duration, retinopathy status, medication history, and explicit adjudication of non‐DKD. Mechanistically, the phenotype is thought to reflect a predominantly tubulointerstitial and microvascular pathway rather than the classical glomerular one, consistent with an acute‐to‐chronic transition driven by tubular rather than podocyte injury.^18^ Because albuminuria‐based screening is blind to this pathway, a meaningful subset of patients is identified late or not at all.^12^, ^14^

Moreover, progression is heterogeneous even among albuminuric patients: latent‐class analyses separate stable patients from rapid progressors, and biopsy series show that only a subset of people with T2D display classical diabetic glomerulopathy, the remainder exhibiting tubulointerstitial, vascular, or mixed lesions.^3^, ^9^ This heterogeneity motivates moving beyond aggregate functional markers toward cell‐state‐resolved readouts, and its biological basis is now well enough defined to make that case concrete. Chronic hyperglycaemia converges on a unifying pathway in which mitochondrial overproduction of superoxide activates the polyol, hexosamine, protein kinase C, and advanced glycation end‐product routes, as Brownlee established, so that one insult diversifies into multiple programs.^19^ Mitochondrial dysfunction in tubular epithelium, reviewed by Forbes et al.,^20^ links this stress to the energy failure of the injured proximal tubule, whereas transforming growth factor beta (TGF‐β) signaling, identified by Meng et al.^21^ as the master regulator of renal fibrosis, drives the interstitial matrix‐producing phenotype.

These programs are not distributed evenly across the kidney. Diabetic injury affects both glomerular and tubulointerstitial compartments, yet tubulointerstitial damage correlates most strongly with progression.^22^, ^23^ On that basis, Vallon et al.^24^ proposed a tubular hypothesis in which proximal tubule hyperreabsorption and growth drive early hemodynamic and structural change, casting the tubule as initiator rather than bystander. Consistent with this, Nair et al.^25^ linked structural lesions to molecular and functional outcomes by molecular morphometry, and Pavkov et al.^26^ showed that tubular injury markers track advanced disease. At the cell‐type level, podocyte depletion underlies the glomerular pathway, whereas maladaptive proximal tubule responses, including a proinflammatory secretory program characterized by Wu et al.,^27^ drive the tubulointerstitial one. Genetic susceptibility further shapes which pathway predominates,^28^ and frequent coexistence of diabetic and nondiabetic kidney disease complicates classification by clinical features alone.^23^, ^29^ Experimentally, models combining hyperglycaemia with renin‐dependent hypertension reproduce the metabolic‐hemodynamic synergy seen clinically, indicating concurrent mechanisms.^30^ This diversity, recognized early by Susztak et al.^31^ as grounds for personalized management, is what an aggregate marker obscures and a cell‐state readout would expose.

Efforts to do better have concentrated on circulating and urinary protein biomarkers, building on the DKD consensus definitions of Tuttle et al.^10^ Niewczas et al.^32^ defined a circulating inflammatory signature anchored on tumour necrosis factor receptors (TNFRs) that predicts progression to kidney failure, extending observations by Gohda et al.^33^ and Niewczas et al.^34^ that circulating TNFR1 and TNFR2 predict end‐stage disease in both T1D and T2D, and consistent with plasma associations reported by Coca et al.^35^ Building on these analytes, KidneyIntelX combines plasma TNFR1, TNFR2, and kidney injury molecule‐1 (KIM‐1) with clinical variables in a random forest; Chan et al.^36^ validated it with an area under the curve (AUC) near 0.77, exceeding clinical models near 0.62. Likewise, the Kidney Failure Risk Equation (KFRE) of Tangri et al.^37^, ^38^ predicts kidney failure with a concordance statistic near 0.90 and anchors current guidelines. In parallel, urinary proteomic and peptidomic classifiers, exemplified by the CKD273 signature of Good et al.^39^ and evaluated in the PRIORITY trial by Tofte et al.,^40^ anticipate progression before albuminuria becomes overt. These tools represent progress, yet each reports risk as a scalar or fixed panel without resolving which cellular processes, glomerular or tubular, immune or fibrotic, are active, and so cannot guide mechanism‐matched therapy or capture endotype.

These limitations frame the proposition we advance here, that urine is an underused route to cellular and spatial resolution in DKD. As shown in Figure 1, urine integrates signals along the entire nephron and simultaneously carries shed cells, EVs, and cfDNA from defined kidney compartments.^41^, ^42^, ^43^ We term the integrated reading of these three compartments the digital urinary biopsy: their joint molecular content is anchored to spatial single‐cell atlases of the kidney to infer, without a needle, the active cell states of the diseased organ. The conceptual advance is the anchoring step. Whereas prior accounts of the liquid kidney biopsy catalogued individual analytes,^44^, ^45^ we formalize the mixed, degraded urinary signal as an observation to be inverted against a spatial cellular reference, so that atlas‐anchored molecular profiling becomes an ill‐posed inverse problem: recovery of per‐cell‐state composition and niche‐associated program activity from a noisy biofluid measurement, regularized by an atlas prior. The objective of this narrative review is to establish that framework and define what its validation requires. We accordingly assess why urine is an information‐rich window onto the kidney and how it compares with tissue and blood, examine each molecular pillar, survey the anchoring atlases, formalize the inverse problem and its computation, and specify biomarker qualification, the endotype‐guided use case, and the obstacles separating concept from validated clinical utility.

**FIGURE 1:**
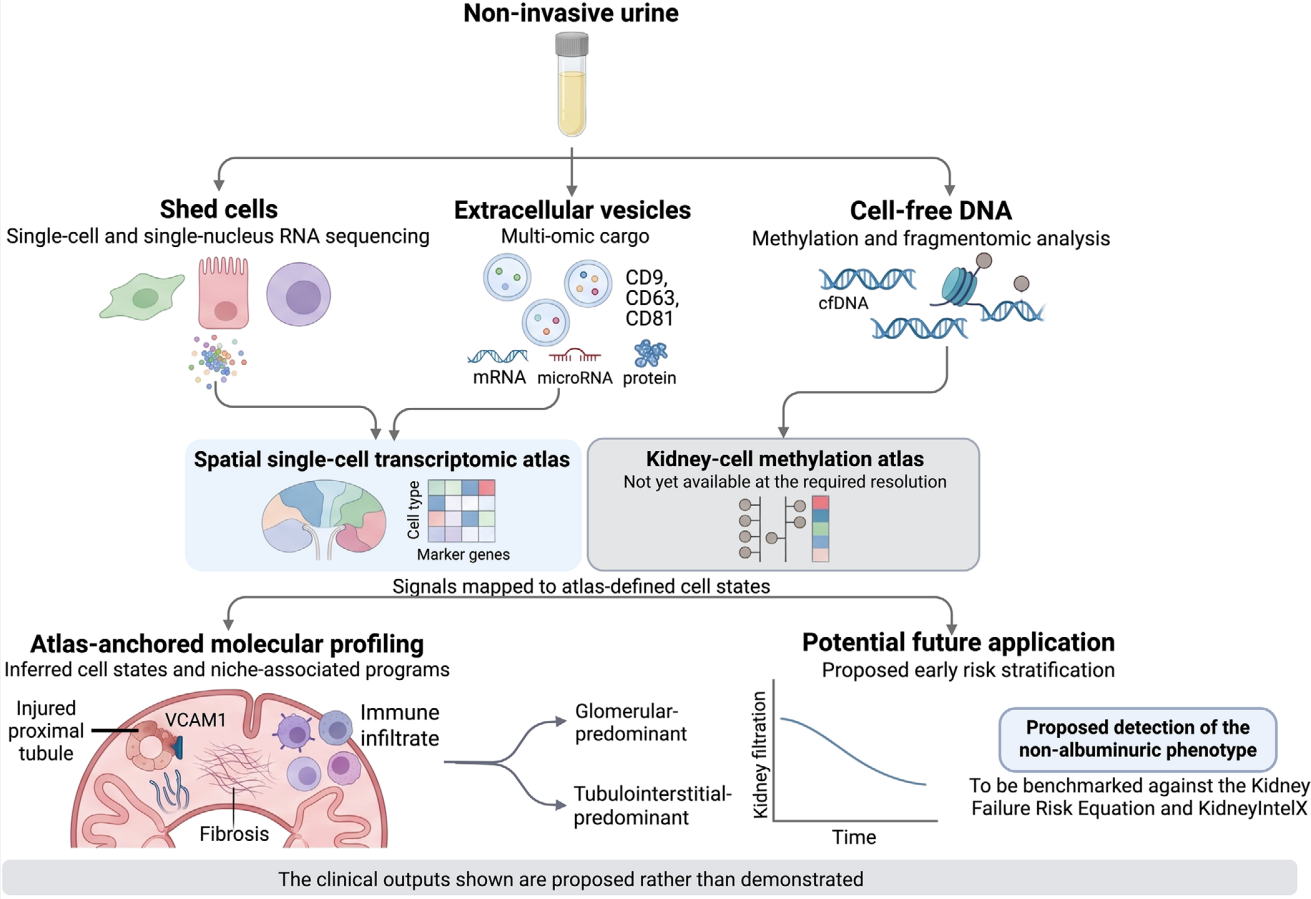
The digital urinary biopsy concept. Schematic overview of the integrated framework. Urine collected non‐invasively is partitioned into three molecular compartments, namely shed cells, EVs, and cfDNA. Each compartment is profiled by an appropriate assay (single‐cell or single‐nucleus RNA sequencing for cells, multi‐omic cargo analysis for vesicles, and methylation and fragmentomic analysis for cfDNA). The resulting signals would be computationally anchored to spatial single‐cell reference atlases of the kidney, with the aim of inferring active cell states and niche‐associated molecular programs to support endotype assignment and early risk stratification, including detection of the non‐albuminuric phenotype. Arrows indicate the analytical flow from biofluid to potential future clinical application. The clinical outputs shown are proposed rather than demonstrated. Created with BioRender: https://app.biorender.com/illustrations/6ab383aa71688bf75c17d7ae.

## 2 URINE AS AN INFORMATION‐RICH WINDOW ONTO THE NEPHRON

The rationale for a urinary readout rests on a simple anatomical fact. Urine is the only biofluid that has passed through every nephron segment, from the glomerular filtration barrier through the proximal tubule, the loop of Henle, the distal convoluted tubule, and the collecting duct, before exiting through the urinary tract.^41^ As it transits, it acquires cellular and molecular cargo from each of these segments, so that the composition of urine reflects, in aggregate, the state of the renal parenchyma.^41^, ^46^ Blood, by contrast, samples the systemic compartment, so kidney‐derived material is only a small fraction of what is measured.^47^, ^48^ The case for urine, therefore, is one of anatomical proximity and signal enrichment, and it has motivated the longstanding ambition of a non‐invasive liquid kidney biopsy.^44^, ^45^ The molecular content of urine that supports this ambition can be grouped into three compartments (Figure 2). First, urine carries intact and viable cells that have detached from the tubular epithelium and the glomerulus, together with infiltrating immune cells and cells of the lower urinary tract.^43^, ^49^ Second, it contains EVs, including exosomes and microvesicles, that bud from the apical membranes of every nephron segment and enclose RNA, protein, and lipid cargo reflective of the parent cell.^41^, ^46^ Third, it contains cell‐free nucleic acids, principally cfDNA derived from dying cells of the kidney and urinary tract, whose epigenetic marks retain the identity of the cell of origin.^42^, ^50^ These compartments are complementary rather than redundant. Shed cells provide the deepest single‐cell molecular phenotype but are sparse and fragile;^43^, ^49^ vesicles are abundant and stable but report cargo selectively sorted by the parent cell;^41^, ^51^ and cfDNA is robust and quantitative but coarse in cellular resolution unless interpreted through methylation.^48^, ^52^ Considered together, the three offer more than any one alone.

**FIGURE 2:**
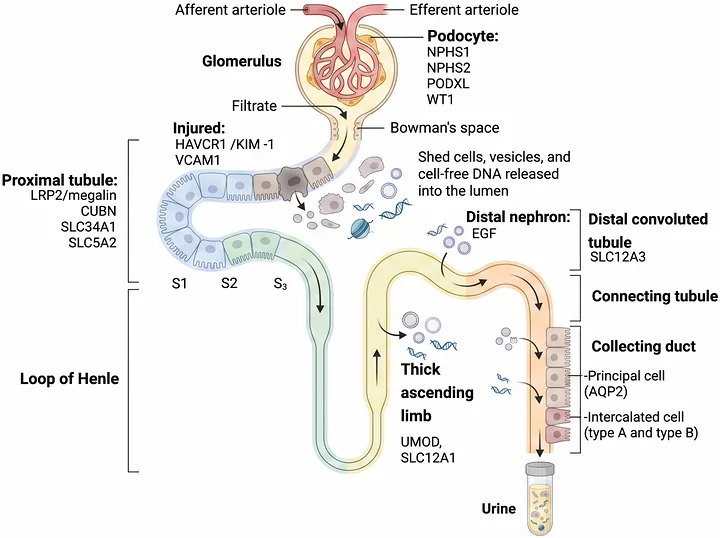
The nephron as a source of urinary biomarkers. Anatomical map of the nephron, from the glomerulus through the proximal tubule, loop of Henle, distal convoluted tubule, and collecting duct, annotated with the cell‐of‐origin markers that allow urinary signals to be traced to specific segments. Podocyte signals (for example NPHS1 and WT1), proximal tubule signals (for example SLC34A1, CUBN, and the injury markers HAVCR1 and VCAM1), and distal nephron signals (for example EGF) are indicated alongside the corresponding shed cells, vesicles, and cfDNA fragments released into the tubular lumen. The figure conveys the principle that urine integrates segment‐resolved information from the entire nephron. Created with BioRender: https://app.biorender.com/illustrations/6a4601fc47bffe46834a4e35.

It is useful to situate the digital urinary biopsy against the two established routes to molecular information about a diseased kidney, namely the tissue biopsy and the blood‐based liquid biopsy (Figure 3). The percutaneous kidney biopsy remains the diagnostic reference, yet it samples only a few glomeruli from a single cortical site and carries procedural risk: across 118 064 native biopsies, haematoma occurred in 11% of patients, bleeding requiring transfusion in 1.6%, and death in 0.06%.^53^ It therefore cannot be repeated often enough to track disease activity over time. The blood‐based liquid biopsy, by contrast, is repeatable and minimally invasive, and the heritage of circulating tumour DNA analysis in oncology, reviewed by Wan et al.,^47^ established the statistical and preanalytical foundations on which kidney applications now build. Blood, however, dilutes kidney‐derived signals within a systemic pool dominated by hematopoietic contributions, which constrains sensitivity to organ‐specific cell states.^48^ Urine occupies a complementary position. It is sampled non‐invasively and repeatedly like blood, yet it is anatomically enriched for kidney‐derived material like the biopsy, and it uniquely preserves intact cells alongside vesicles and cfDNA in a single specimen.^41^, ^43^, ^54^ The accompanying trade‐off is that urinary material is sparser, more degraded, and admixed with lower urinary tract contributions,^43^, ^49^, ^55^ which is precisely the problem that atlas anchoring is designed to solve. Considered in this comparative frame, the digital urinary biopsy is best understood not as a replacement for tissue or blood but as a third modality whose distinctive value is longitudinal, cell‐resolved, organ‐enriched sampling. That urine reports renal pathophysiology is not a new idea, since established urinary biomarkers already exploit it. KIM‐1 and neutrophil gelatinase‐associated lipocalin report proximal tubule stress, monocyte chemoattractant protein‐1 reports interstitial inflammation, and epidermal growth factor reports the integrity of the distal nephron, with a low urinary epidermal growth factor concentration predicting progression.^56^ Indeed, Ju et al.^57^ used a tissue transcriptome‐driven strategy to nominate urinary epidermal growth factor as a marker of CKD progression, anchoring a urinary protein directly to a renal transcriptomic source. Likewise, Schrauben et al.^58^ and Nowak et al.^59^ showed that tubular injury markers measured in plasma predict progression in diabetes beyond albuminuria. The urinary evidence for the same markers is weaker than is often assumed. Among 260 Pima Indians with T2D followed for a median of 14 years, urinary neutrophil gelatinase‐associated lipocalin was associated with ESKD, yet adding it to albuminuria and filtration rate raised the concordance statistic only from 0.828 to 0.833, an increment the investigators judged unlikely to be clinically meaningful.^60^ That single analytes measured in urine add so little to standard markers is itself part of the case for a cell‐resolved readout. Taken together, these findings establish the principle that urinary molecules track defined renal processes. What they have lacked, however, is cellular resolution; each marker reports an aggregate that cannot, on its own, be assigned to a specific cell state or microanatomic niche. The digital urinary biopsy concept extends this established principle by replacing single analytes with compartment‐wide molecular profiles and by adding the anchoring step that assigns those profiles to cell states. The three pillars that make this possible are considered next, and the evidence underlying each is graded study by study in Table 1.

**FIGURE 3:**
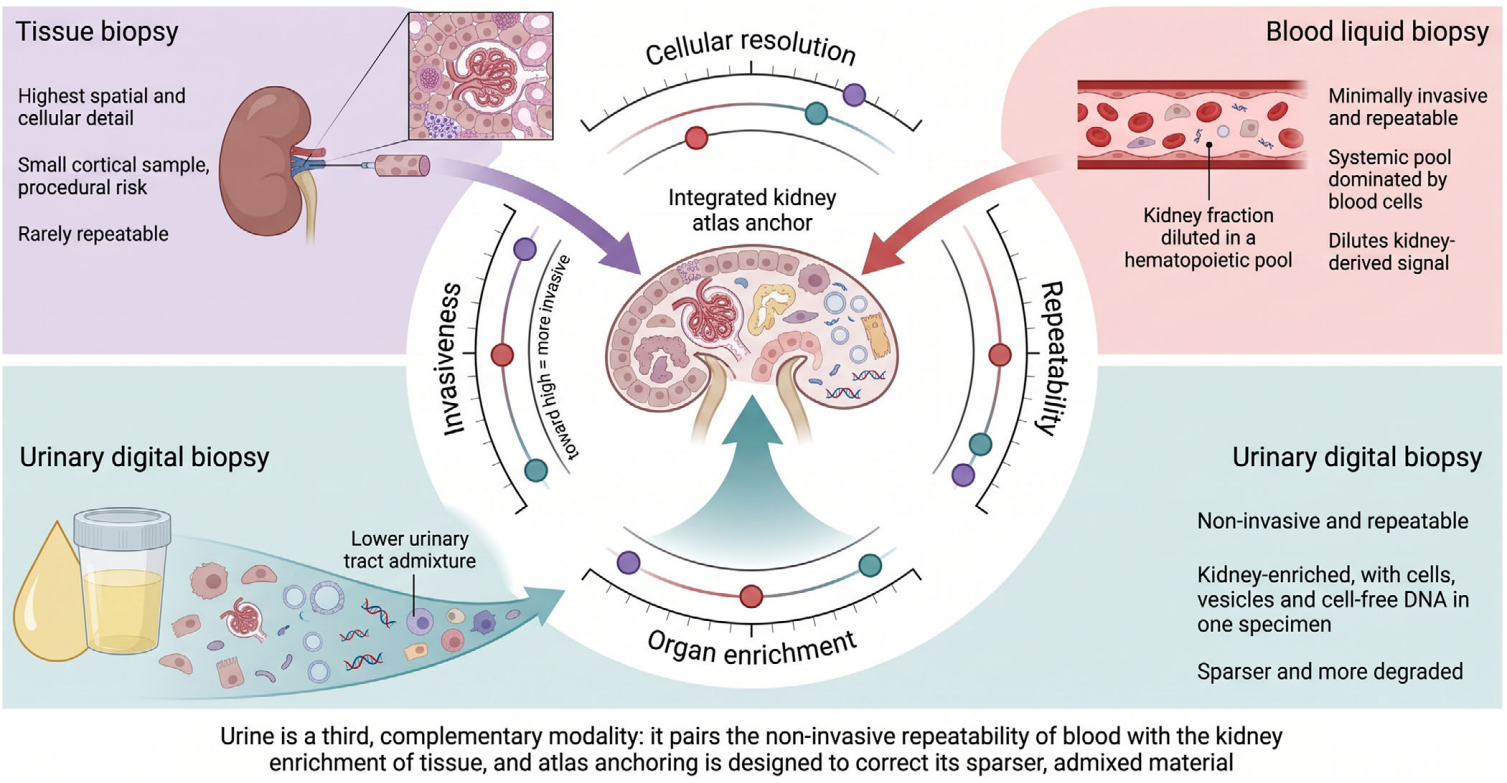
Comparative information content across kidney interrogation modalities. Conceptual comparison of the three routes to molecular information about a diseased kidney, namely the tissue biopsy, the blood‐based liquid biopsy, and the urinary digital biopsy, arranged along axes of invasiveness, repeatability, organ enrichment, and cellular resolution. The tissue biopsy provides the highest spatial and cellular detail but samples a small cortical region, carries procedural risk, and is rarely repeatable. The blood liquid biopsy is minimally invasive and repeatable but dilutes kidney‐derived signal within a systemic, haematopoietically dominated pool. The urinary digital biopsy combines non‐invasive, repeatable sampling with anatomical enrichment for kidney‐derived cells, vesicles, and cfDNA, at the cost of sparser and more degraded material admixed with lower urinary tract contributions. The figure positions the urinary modality as complementary to, rather than a replacement for, tissue and blood. Created with BioRender: https://app.biorender.com/illustrations/6a462bf893d76312b9b3123c.

**TABLE 1:** Evidence base underlying the three urinary compartments.

Pillar	Study	Population and design	Participants and samples	Specimen handling and assay	Outcome	Reported performance	Validation status	Limitations
**I. Shed cells**	Abedini^43^	Mixed kidney disease and a pooled healthy reference; cross‐sectional feasibility	5 subjects, 17 urine samples (spot and 24‐hour), 23,082 cells	Fresh urine, centrifugation; droplet‐based scRNA‐seq	Almost all kidney cell types recoverable; composition subject‐specific and stable across collection method and time	Descriptive; no diagnostic metric reported	Reference dataset; no independent clinical validation	5 subjects; cells are not independent patient‐level observations; lower urinary tract cells abundant; no DKD‐specific endpoint
**I. Shed cells**	Suthanthiran^65^	Kidney allograft recipients; multicentre prospective (CTOT‐04)	485 recipients, 4300 urine specimens	Urinary cell pellet; RT‐qPCR of CD3e mRNA, CXCL10 mRNA, 18S rRNA	Three‐gene signature diagnoses and anticipates acute cellular rejection	AUC 0.85 (95% CI 0.78–0.91); external validation AUC 0.74 (0.61–0.86)	Externally validated within transplantation	Targeted qPCR panel, not cell‐state resolved; allograft rejection, not native diabetic disease
**I. Shed cells**	Latt^66^	Focal segmental glomerulosclerosis; single centre	12 subjects, 17 urine samples captured as 23 cell samples	Urine sediment; droplet‐based scRNA‐seq	Monocyte subsets and podocyte epithelial‐to‐mesenchymal transition signatures identified	Signature comparison only; no accuracy metric	Compared with NEPTUNE biopsy transcriptomes; not prospectively validated	Small cohort; FSGS rather than DKD; no patient‐level diagnostic estimate
**I. Shed cells**	Klocke^49^	Acute kidney injury; single centre	32 patients, 40 urine samples, 42 608 cells	Flow‐cytometric sorting of viable cells before droplet‐based scRNA‐seq	Injury and repair states of tubular epithelium; adaptive progenitor‐like cells expand during regeneration	Descriptive; no accuracy metric	Compared with post‐mortem AKI biopsies and published mouse data	AKI rather than DKD; sorting pipeline not widely replicated; transfer of findings across diseases untested
**I. Shed cells**	Ma^69^	Early (eGFR at least 30) and advanced (eGFR below 30) DKD; single centre	8 patients (4 early, 4 advanced); 7248 cells captured and 4,314 retained after quality control (530 early, 3748 advanced); pooled urine from 10 healthy individuals as external control	150–200 mL morning urine, centrifugation, erythrocyte lysis, dead‐cell removal; multiplexed scRNA‐seq in two batches	Most urinary tubular cells occupy adaptive states; ratio of progenitor and proliferative to fibrosis‐committed cells reports tubular injury; MAF implicated in fibrosis fate	No diagnostic accuracy metric reported	Reannotation of public AKI, DKD, and FSGS urine datasets; no independent clinical cohort	Authors state the sample is small; data not adjusted for eGFR; adaptive markers also enriched in urogenital epithelium; the two arms differ sevenfold in retained cell number
**I. Shed cells**	Chen^84^	DKD; retrospective analysis of public single‐cell and bulk transcriptomic repositories	2089 urine‐derived cells plus public bulk cohorts	In silico separation of kidney‐origin urinary cells; machine‐learning feature selection	Three‐gene panel (PDK4, RHCG, FBP1); injured proximal tubule cells upregulate HAVCR1 and VCAM1	AUC above 0.9 in the reported datasets	Validated in independent public datasets only	No prospectively collected specimens and no analytical assay validation; the panel is also downregulated across several other chronic kidney diseases, which limits specificity
**I. Shed cells**	Arazi^76^	Lupus nephritis; multicentre, paired kidney and urine	Paired kidney and urine single‐cell profiling; 21 leukocyte subsets defined	Kidney biopsy and urine; droplet‐based scRNA‐seq	Immune cell gene expression in urine correlates with that in kidney	Correlative; no accuracy metric	Not validated as a diagnostic test	Lupus rather than diabetes; immune compartment only; no demonstration that epithelial cell states transfer in the same way
**II. EVs**	Pisitkun^41^	Healthy adults; discovery proteomics	Pooled and individual urine specimens	Ultracentrifugation; mass spectrometry	Urinary exosomes originate from cells lining the entire nephron and urinary space	Descriptive	Foundational and widely replicated	No disease endpoint; ultracentrifugation is not a clinical workflow
**II. EVs**	Dwivedi^93^	T1D with varying kidney involvement; discovery with replication in type 1 and T2D	Approximately 200 urinary EV mRNA samples	Vesicle isolation from clinical urine; genome‐wide RNA sequencing	Vesicular transcriptome resembles the kidney transcriptome (more than 10 000 mRNAs); 13 upregulated proximal tubule genes; six‐gene stress score	Stress score reflected long‐term decline in kidney function and identified normoalbuminuric early decliners; no AUC reported	Replicated in independent diabetes cohorts	Observational; no prespecified threshold; preanalytical workflow not standardized across sites
**II. EVs**	Barutta^94^, Delic^95^, Lv^96^, Jia^97^, Ghai^99^	Candidate microRNA studies in type 1 and T2D	Small cohorts; Delic et al. profiled 8 controls, 8 with T2D, and 8 with diabetic nephropathy, with a separate confirmation cohort	Ultracentrifugation or precipitation; microarray, small‐RNA sequencing, or RT‐qPCR	Individual microRNAs altered in DKD, including miR‐145, miR‐130a, miR‐155, miR‐424, miR‐29c, miR‐320c, miR‐30d/30e, and miR‐192/194/215	Fold‐change associations; diagnostic accuracy rarely reported	Little cross‐study replication; platforms and normalization differ	Associative rather than validated; preanalytically heterogeneous; the reported direction of change is not consistent across studies for several microRNAs
**II. EVs**	Kalani^103^	T2D with and without proteinuria	Single‐centre cohort	Urinary exosome preparation; immunoblot for Wilms tumor‐1	Wilms tumor‐1 detectable in urinary exosomes and higher in patients with declining function	Semi‐quantitative; no accuracy metric	Not independently validated	Immunoblot readout without a normalization standard; small cohort; podocyte specificity not established in urine
**II. EVs**	MISEV and ISEV Urine Task Force^101^, ^108^, ^109^; Salih^110^	Consensus reporting standards and quantitative immunoassay development	Not applicable	Defines collection, isolation, characterization, and reporting requirements	Preanalytical and reporting standards for urinary vesicles; normalization remains unresolved	Not applicable	Consensus guidance rather than outcome data	Most diabetic vesicle studies predate or do not meet these standards, which limits cross‐study comparison
**III. cfDNA**	Moss^48^	Plasma cfDNA in health and multiple diseases	Reference atlas of 25 human tissues and cell types	Methylation array; non‐negative least squares deconvolution	Plasma cfDNA of healthy donors derives from leukocytes (55%), erythrocyte progenitors (30%), endothelium (10%), and hepatocytes (1%)	Validated in silico and against in vitro DNA mixtures of known proportion	Analytically validated for plasma	Plasma rather than urine; kidney is not resolved at cell‐type or cell‐state level
**III. cfDNA**	Loyfer^117^	Normal human cell types; reference resource	Whole‐genome methylation atlas at higher resolution than prior atlases	Whole‐genome bisulfite sequencing of sorted cell types	Provides the reference granularity needed to separate closely related cell states	Not applicable	Reference resource	Tissue‐level and not yet applied to urinary cfDNA; kidney cell states of interest are not separately represented
**III. cfDNA**	Li^118^	Plasma cfDNA; deep‐learning deconvolution (cfSort)	Atlas of 521 non‐cancer tissue samples spanning 29 major tissue types	Fragment‐level methylation features; supervised deep learning	Sensitive and accurate tissue deconvolution of plasma cfDNA	Superior sensitivity and accuracy to existing deconvolution methods on benchmark data	Benchmarked, with two clinical use cases demonstrated	Plasma; 29 tissues rather than kidney cell types; not evaluated on urinary cfDNA
**III. cfDNA**	Bloom^123^	Kidney allograft recipients; prospective, biopsy‐matched (DART)	102 recipients, 107 biopsy‐matched specimens	Plasma donor‐derived cfDNA	Discriminates active rejection from absence of rejection	AUC 0.74 (95% CI 0.61–0.86) for any rejection and 0.87 for antibody‐mediated rejection; at a 1.0% threshold, positive predictive value 61% and negative predictive value 84%	Prospective and biopsy‐matched	Plasma; relies on donor‐recipient genotype contrast, which has no counterpart in native kidney disease; positive predictive value modest
**III. cfDNA**	Aubert^124^	Kidney allograft recipients; observational population‐based study across 14 centres in Europe and the United States	2882 recipients enrolled; primary analysis 1134; external validation 1748, including 439 African American recipients	Plasma donor‐derived cfDNA	Adds discrimination for rejection beyond standard‐of‐care monitoring	AUC improved from 0.777 (95% CI 0.741–0.811) to 0.821 (0.784–0.852), *p* = .0011	Externally validated in independent cohorts	Plasma and transplantation, with genotype‐based ground truth; does not transfer to cell‐state inference in native DKD
**III. cfDNA**	Botezatu,^54^ Burnham,^125^ Cheng^126^	Transrenal and urinary cfDNA in transplantation and urinary tract infection	Single‐centre and small multicentre cohorts	Urinary cfDNA extraction; single‐stranded library preparation to recover short and degraded fragments; sequencing	Urinary cfDNA is recoverable and carries donor‐derived and microbial signal	Diagnostic for infection and donor‐derived signal; no kidney cell‐state metric	Not validated for kidney cell‐state inference	Urinary cfDNA is contributed substantially by lower urinary tract epithelium; no kidney‐cell‐resolved methylation reference exists; native DKD unexplored

*Note*: For each pillar the table records the population studied, the number of participants and samples, specimen handling and assay, principal outcome, reported performance, validation status, and principal limitations. The final column records null, non‐replicated, and cross‐disease evidence alongside positive findings.

Abbreviations: AKI, acute kidney injury; AUC, area under the curve; cfDNA, cell‐free DNA; CI, confidence interval; CTOT, Clinical Trials in Organ Transplantation; DKD, diabetic kidney disease; eGFR, estimated glomerular filtration rate; EVs, extracellular vesicles; FSGS, focal segmental glomerulosclerosis; ISEV, International Society for Extracellular Vesicles; MISEV, minimal information for studies of extracellular vesicles; NEPTUNE, Nephrotic Syndrome Study Network; RT‐qPCR, reverse transcription quantitative polymerase chain reaction; scRNA‐seq, single‐cell RNA sequencing; T1D, type 1 diabetes; T2D, type 2 diabetes

### 2.1 Pillar I: Shed cells and urinary single‐cell transcriptomics

The most direct way to read kidney cell states from urine is to sequence the cells themselves. The technical substrate for this is the maturation of droplet‐based single‐cell RNA sequencing, introduced through nanoliter‐droplet barcoding by Macosko et al.^61^ and scaled commercially in the platform characterized by Zheng et al.,^62^ which together made transcriptome‐wide profiling of thousands of individual cells routine. The complementary development of single‐nucleus methods by Habib et al.^63^ extended the approach to archived and difficult‐to‐dissociate tissue, an advance directly relevant to fragile urinary material, and the coordinating ambition of the Human Cell Atlas articulated by Regev et al.^64^ established the reference‐atlas paradigm on which the anchoring logic of this review depends.

On this methodological foundation, the feasibility of urinary application was established by Abedini et al.,^43^ who applied droplet‐based single‐cell RNA sequencing to urine and recovered 23 082 cells across 17 samples from 5 subjects, capturing nearly the full diversity of kidney cell types, including podocytes, proximal tubule, loop of Henle, and collecting duct, alongside immune and lower urinary tract cells, with subject‐specific and reasonably stable cellular composition. The principle that urinary cell RNA reports kidney pathology had in fact been established earlier and at scale in transplantation, where Suthanthiran et al.^65^ showed in a multicentre study that a three‐gene messenger RNA signature measured in urinary cells diagnoses and even anticipates acute cellular rejection of kidney allografts, providing the canonical demonstration that cells shed into urine carry actionable information about the state of the parenchyma. Building on that proof of principle, the urinary single‐cell study of Abedini et al. provided feasibility evidence that the major kidney cell types can be recovered from urine sediment and read at single‐cell resolution, rather than evidence that urinary shedding is representative, since the 23 082 cells derive from 5 subjects and cells within a subject are not independent patient‐level observations.^43^ Correspondingly, Latt et al.^66^ established urinary single‐cell sequencing in focal segmental glomerulosclerosis, identifying monocyte subpopulations and podocyte signatures of epithelial‐to‐mesenchymal transition that tracked disease activity. Together, these reports demonstrated that urinary single‐cell transcriptomics is biologically informative as well as technically possible. Subsequent work extended the approach to acute and chronic injury and refined its cellular interpretation. Klocke et al.^49^ built a flow‐sorting pipeline that enriched viable urinary cells before sequencing and generated 42 608 transcriptomes from 40 samples of 32 patients with acute kidney injury, defining injury and repair states of the tubular epithelium and identifying adaptive, progenitor‐like cells that expand during regeneration. This is significant because the same maladaptive and failed‐repair states recur across kidney diseases and are central to the diabetic context.^67^, ^68^ In DKD specifically, Ma et al.^69^ profiled urine sediment from patients with early and advanced disease and found that most urinary tubular cells occupy an adaptive state, which they designated adaptive tubular epithelial cells, marked by epithelial cell adhesion molecule, OCIAD2, and the small heat‐shock protein CRYAB, and resolvable into several subtypes. Crucially, the ratio of progenitor‐like and proliferative adaptive cells to fibrosis‐committed adaptive cells carried diagnostic information about tubular injury, and the transcription factor MAF was implicated in the fate decision toward fibrosis, with immunofluorescence showing a shift in MAF localization across disease stages.^69^

Mechanistically, these failed‐repair states are profibrotic for a definable reason. Yang et al.^70^ showed that injured epithelial cells arrest in the G2/M phase of the cell cycle and, from that arrested state, secrete TGF‐β and connective tissue growth factor that drive interstitial fibrosis, which explains why an accumulation of adaptive rather than resolved tubular cells forecasts scarring. Furthermore, the same injured cells activate a partial epithelial‐to‐mesenchymal transition, since Lovisa et al.^71^ and Grande et al.^72^ demonstrated that Snail‐ and Twist‐driven mesenchymal programs, without full loss of epithelial identity, induce cell‐cycle arrest, dedifferentiation, and loss of intercellular adhesion in renal fibrosis. That loss of adhesion is, in turn, the plausible mechanism by which such cells detach from the basement membrane and enter the urinary space,^71^, ^72^ which connects the molecular biology of injury directly to the sampling process on which this pillar depends. Notably, even the canonical injury marker reflects mechanism rather than mere correlation, because KIM‐1, shown by Ichimura et al.^73^ to be a phosphatidylserine receptor that confers a phagocytic phenotype on surviving tubular cells, is upregulated on injured epithelium and released into urine after ectodomain cleavage, so its urinary appearance reports an active clearance program rather than passive leakage. These observations indicate that urinary cells report the mechanistic trajectory of tubular fate rather than only the presence of injury, which is precisely the kind of cell‐state information that aggregate biomarkers cannot supply. This evidence should nonetheless be read with the diseases in which it was generated. Acute kidney injury, lupus nephritis, focal segmental glomerulosclerosis and transplantation establish biological plausibility, and the only dedicated diabetic study profiled 8 patients,^69^ so representative shedding in DKD remains to be demonstrated.

The specificity of these cell states rests on genetic evidence rather than association alone, which matters because a urinary readout is only as meaningful as the causal standing of the states it reports. Conditional deletion of Twist1 or Snai1 in proximal tubular epithelium inhibited the partial epithelial‐to‐mesenchymal programme, preserved epithelial integrity, restored proliferation and dedifferentiation‐associated repair, and attenuated interstitial fibrosis;^71^ reactivation of Snai1 in renal epithelium proved to be required for fibrosis to develop, and established disease was reversed when Snail1 expression was targeted.^72^ Independently, cell‐cycle arrest in G2/M was shown to mediate fibrosis after injury rather than to accompany it,^70^ and genetic fate tracing established that myofibroblasts derive from resident pericytes and interstitial fibroblasts rather than from epithelial transdifferentiation,^74^ a conclusion since extended to human kidney fibrosis.^75^ Lineage‐resolved single‐nucleus profiling then showed that failed‐repair proximal tubule states persist into chronic disease rather than resolving.^67^, ^68^ These experiments establish that the states this framework proposes to read are causally implicated in progression. They do not establish that the abundance of such cells in urine is proportional to their abundance in tissue, which is a question of sampling rather than of biological specificity.

The immune compartment of urine is similarly informative, and here lessons from lupus nephritis are instructive. Within the Accelerating Medicines Partnership, Arazi et al.^76^ showed that immune cells recovered from urine closely resemble those infiltrating the kidney parenchyma, validating urine as a surrogate for the intrarenal immune environment. This correspondence is conceptually important for the anchoring logic developed later, because it implies that at least some urinary populations are faithful reporters of tissue niches rather than artifacts of urinary transit. In transplantation, single‐cell analyses of urine and allograft tissue by Wu et al.^77^ similarly captured inflammatory responses that mirrored biopsy findings. Furthermore, urinary cells include genuine kidney progenitor populations, as shown by single‐cell analyses that identified progenitor cells in human urine with regenerative gene programs,^78^ and they include detached podocytes, since the excretion of viable podocytes in urine, characterized by Vogelmann et al.,^79^ reports glomerular injury and is relevant to detecting the glomerular dimension of disease independently of albuminuria. The recurring theme across these studies is that urine yields a multilineage cellular sample, comprising epithelial, immune, and progenitor cells, whose states can be assigned to known kidney biology.

Methodological reviews by Wu et al.^80^ anticipated this trajectory by setting out the promise of single‐cell sequencing for kidney disease, and the subsequent publication of standardized protocols for constructing urine single‐cell atlases,^81^ together with broader surveys of urine single‐cell transcriptomics across human kidney disease^82^ and of single‐cell genomics in nephrology more generally,^83^ has consolidated the approach into a reproducible workflow. Recent efforts have begun to translate these descriptive findings into classifiers. Machine learning applied to urinary single‐cell data has nominated injured proximal tubule cells expressing hepatitis A virus cellular receptor 1 and vascular cell adhesion molecule 1, together with compact gene panels reported to discriminate DKD with high accuracy, although that study analysed public single‐cell and bulk transcriptomic repositories rather than prospectively collected specimens, reported no patient‐level data splits, and evaluated the panel only in further public datasets, so it is not equivalent to prospective clinical assay validation^84^ (Figure 4).

**FIGURE 4:**
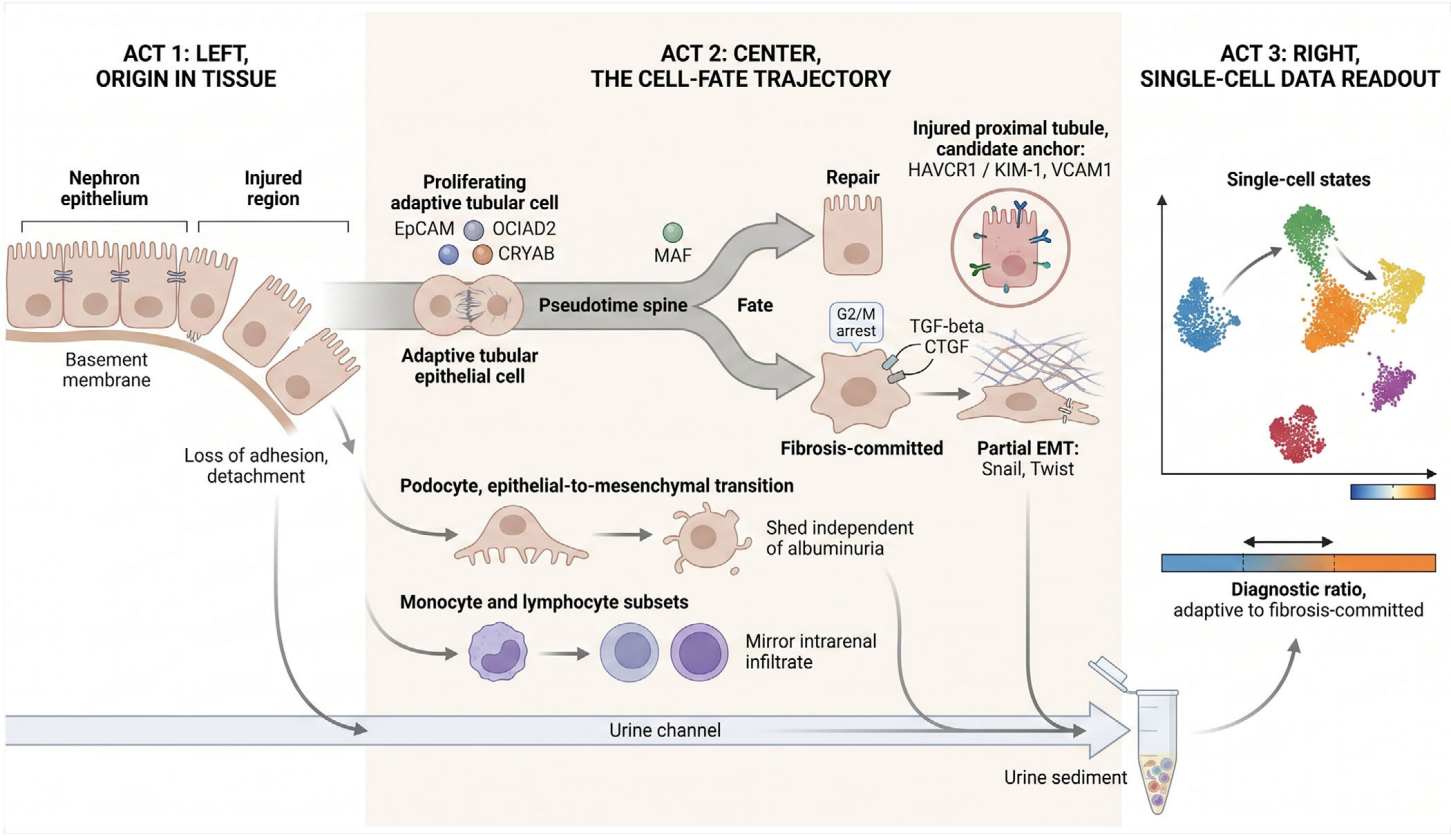
Urinary cell states recoverable by single‐cell sequencing in DKD. Representation of the major cell populations recovered from urine sediment, organized by lineage. Epithelial populations include adaptive tubular epithelial cell states ranging from progenitor‐like and proliferative to fibrosis‐committed, with key markers indicated. Glomerular signals include podocytes with epithelial‐to‐mesenchymal transition programs. Immune populations include monocyte and lymphocyte subsets that mirror intrarenal infiltrates. Injured proximal tubule cells expressing HAVCR1 and VCAM1 are highlighted as candidate anchors. The figure illustrates that urinary sequencing reports cell‐fate trajectories rather than mere injury presence. Created with BioRender: https://app.biorender.com/illustrations/0ef463297206a9bfb0f0b88d.

Nonetheless, two methodological caveats temper enthusiasm. First, urinary cell yield is low and variable, and a substantial fraction of recovered cells are senescent or stressed, with lymphocytes among the most viable and urothelial cells among the most degraded, which biases recovered populations.^43^, ^49^ Second, single‐nucleus sequencing, which Wu et al.^85^ showed reduces dissociation‐induced artifacts and better captures difficult cell types in solid kidney tissue, has not been technically validated in urine. The input material in that setting is not yet defined, since nuclei may be recovered from intact shed cells or from cells already degraded in transit, and performance characteristics on urinary specimens have not been reported. These limitations define the technical frontier for this pillar. Even so, the collective evidence establishes shed‐cell sequencing as the highest‐resolution component of the digital urinary biopsy, providing the cellular phenotypes that the other pillars report only indirectly.

### 2.2 Pillar II: Urinary extracellular vesicles and their multi‐omic cargo

Where shed cells are sparse and fragile, urinary EVs are abundant and comparatively stable, and they carry a curated molecular record of their parent cells.^41^, ^44^, ^46^ The biological premise of this pillar rests on foundational vesicle biology. Valadi et al.^86^ demonstrated that exosomes transfer functional messenger RNA and microRNA between cells, establishing that vesicles carry a transcriptomic payload rather than inert debris, and subsequent syntheses by Raposo and Stoorvogel,^87^ by van Niel et al.,^88^ and by Kalluri and LeBleu^89^ defined the biogenesis, molecular composition, and disease relevance of exosomes and microvesicles, including the selective sorting of cargo that makes vesicle content a reporter of parent‐cell state. Building on this foundation, the renal field began with Pisitkun et al.,^41^ who first identified and proteomically profiled exosomes in human urine and showed that they originate from cells lining the urinary space along the full length of the nephron, establishing urinary vesicles as a window onto every nephron segment. Since then, urinary EVs have been framed explicitly as a route to a liquid kidney biopsy, because their cargo is sorted by the parent cell and therefore reports cell state rather than mere abundance.^46^, ^90^ This selectivity is mechanistically grounded rather than incidental. Exosomes arise as intraluminal vesicles within multivesicular bodies through two convergent routes, namely an endosomal sorting complex required for transport pathway and an ESCRT‐independent route in which ceramide generated by neutral sphingomyelinase drives inward budding, as Trajkovic et al.^91^ demonstrated, while the biogenesis and secretion of these vesicles were systematically mapped by Colombo et al.^92^ Correspondingly, the RNA payload is actively loaded rather than randomly captured, since Villarroya‐Beltri et al.^51^ showed that sumoylated heterogeneous nuclear ribonucleoprotein A2B1 recognizes specific sequence motifs and directs particular microRNAs into exosomes, which explains why the vesicular transcriptome is a curated rather than a representative sample of the cytoplasm. Because these sorting mechanisms are themselves regulated by cell state and stress, the vesicular cargo of an injured proximal tubule cell differs systematically from that of a healthy one,^51^, ^93^ which is the molecular premise that makes vesicles informative reporters. The biogenesis of these vesicles and the diversity of their cargo are depicted in Figure 5.

**FIGURE 5:**
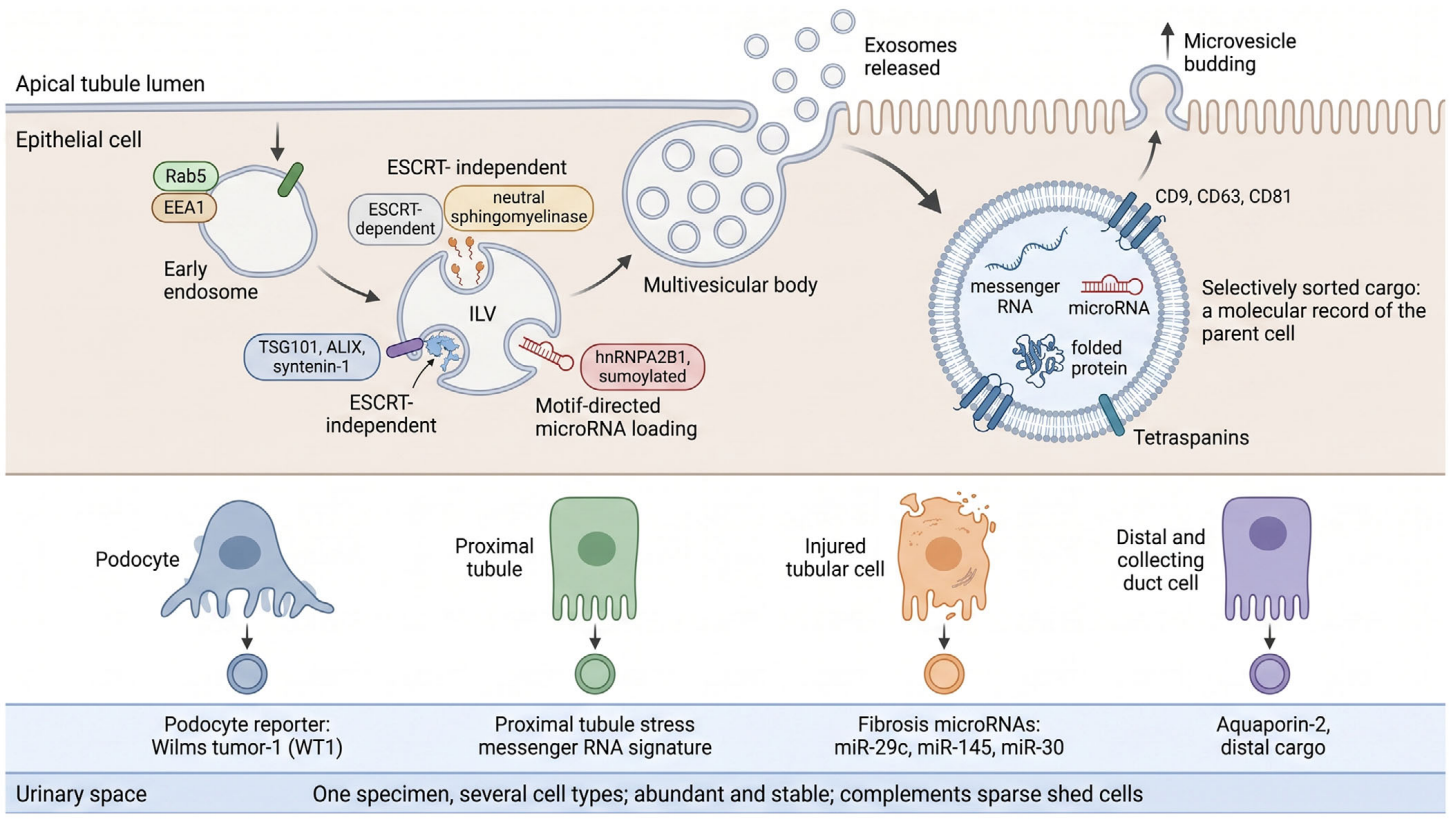
Biogenesis and multi‐omic cargo of urinary EVs. Diagram of vesicle formation, showing exosome release from multivesicular bodies and microvesicle budding from the apical membrane of nephron epithelial cells. The enclosed cargo, comprising messenger RNA, microRNA, and protein, is depicted as a selectively sorted molecular record of the parent cell. Examples relevant to DKD are annotated, including a proximal tubule stress messenger RNA signature, fibrosis‐associated microRNAs, and the podocyte reporter Wilms tumor‐1. The figure conveys why vesicles provide an abundant and cell‐selective complement to sparse shed cells. Created with BioRender: https://app.biorender.com/illustrations/4e5ff4944fbed767b8fe5d17.

The transcriptomic cargo of urinary vesicles has proven particularly informative in diabetes. Dwivedi et al.^93^ performed genome‐wide messenger RNA profiling of urinary EVs and found that the vesicular transcriptome resembles the kidney transcriptome, encompassing more than ten thousand messenger RNAs, and defined a proximal tubule stress signature, spanning roughly a dozen genes, that was upregulated in type 1 DKD and correlated with hyperglycaemia. This is notable because it links a urinary vesicular readout directly to a specific nephron segment and a specific pathophysiologic stimulus, which is the kind of mapping the anchoring framework requires. In parallel, the microRNA cargo of urinary vesicles has yielded candidate markers of early DKD. Barutta et al.^94^ reported altered urinary exosomal microRNAs in incipient diabetic nephropathy, Delic et al.^95^ defined a urinary exosomal microRNA signature in type 2 diabetic nephropathy, and Lv et al.^96^ showed that vesicular microRNA‐29c tracks renal fibrosis. In a separate cohort, Jia et al.^97^ found vesicular microRNA‐192, microRNA‐194 and microRNA‐215 raised in microalbuminuric T2D relative to normoalbuminuric and control subjects, with microRNA‐192 correlating with albuminuria and with TGF‐β 1, although all three fell again in the macroalbuminuric group. Earlier surveys had already established the direction of these effects, with Spanu et al.^98^ showing that urinary exosomes report changes in renal gene and protein expression and Ghai et al.^99^ defining vesicular microRNA profiles associated with diabetic nephropathy in T1D, lending reproducibility to the vesicular microRNA approach.

The most substantial replication to date is that of Barreiro et al.,^100^ who sequenced vesicular microRNAs in T1D and replicated the findings in two independent cohorts and six published datasets spanning both diabetes subtypes and both sexes, with CKD, hypertensive nephropathy, IgA nephropathy, polycystic kidney disease, kidney stones and prostate cancer cohorts as additional controls, 490 individuals in total. Eleven microRNAs were differentially expressed, the strongest evidence being for miR‐192‐5p, miR‐146a‐5p, miR‐486‐5p and miR‐574‐5p, and six further microRNAs were identified as stable normalization references. The panel identified individuals with the fastest decline in kidney function, with sensitivity between 0.75 and 1.00 and specificity between 0.83 and 1.00, and did so within the normoalbuminuric group. MicroRNAs whose abundance varied with urine collection type or with centrifugation before storage had been excluded at the outset, which is the preanalytical discipline this literature has generally lacked.^46^, ^101^ Furthermore, the vesicle proteomics extends this evidence to treatment response. In a randomized crossover trial in which 20 patients with diabetes and albuminuria received telmisartan, empagliflozin, linagliptin and baricitinib in turn, 2441 vesicle proteins were quantified and 355 were associated with albuminuria, while each drug produced a distinct differentially abundant set, of 86, 313, 80 and 47 proteins respectively, with minimal overlap between treatments.^102^ Of the proteins responsive to SGLT2 inhibition, 62% also changed at the transcript level in kidney cells, usually in the same direction, which is among the clearest available demonstrations that a urinary vesicle signal tracks a change occurring within the tissue.

Beyond bulk cargo, vesicles can report injury of specific and otherwise inaccessible cell types, most importantly the podocyte. Kalani et al.^103^ demonstrated that the podocyte transcription factor Wilms tumor‐1 is detectable in urinary exosomes and that its abundance rises in diabetic patients with declining function, providing a non‐invasive readout of podocyte stress. This matters because podocytes are terminally differentiated, are lost early in diabetic glomerular injury, and are difficult to interrogate without biopsy,^79^, ^104^ so a vesicular podocyte reporter addresses a genuine gap. Correspondingly, vesicular markers of distal nephron and collecting duct function have been described,^90^ and Gildea et al.^105^ demonstrated functional exosomal transfer from proximal tubule to distal nephron cells, extending segment‐specific coverage along the nephron and indicating that vesicles also mediate intratubular communication. The collective implication is that urinary vesicles can, in principle, report the activity of several distinct kidney cell types in a single specimen. These strengths are accompanied by real constraints, however, and the field has responded with standardization efforts. Vesicle isolation methods, including ultracentrifugation and precipitation, differ in yield and purity, and urinary vesicle preparations are sensitive to collection conditions, centrifugation protocols, protease and nuclease activity, and the choice of normalization reference, all of which complicate cross‐study comparison.^46^, ^101^ In addition, the measurement also varies with the state of the patient and not with the assay alone. Vesicle concentration tracks urine creatinine closely, and water loading alters both vesicle size and the interference of Tamm‐Horsfall protein with particle counting,^106^ while vesicle excretion scales with nephron mass, correlating with eGFR and total kidney volume, differing by sex, and falling after nephrectomy by far less than the loss of filtration would predict.^107^ Comparison of urinary vesicle measurements between individuals therefore requires an explicit normalization strategy together with a measure of nephron mass or excretion rate.^107^

To address this, the International Society for Extracellular Vesicles issued minimal information guidelines, in successive iterations, and a dedicated position paper on urinary vesicles through its Urine Task Force, defining preanalytical and reporting standards.^101^, ^108^, ^109^ We follow that nomenclature here, using extracellular vesicles as the generic term and reserving exosome for vesicles of demonstrated endosomal origin; where cited studies described their material as exosomal without establishing biogenesis, those preparations are more accurately small extracellular vesicles. Quantitative immunoassays for urinary vesicles, developed by Salih et al.,^110^ have begun to address the normalization problem that has long undermined cross‐study comparison, and the broader recognition that vesicles report systemic as well as local inflammation, observed in autoimmune disease, situates urinary findings within a wider biological context.^111^ Consequently, although the biological case for vesicles as cell‐state reporters is strong, much of the existing DKD literature rests on small, preanalytically heterogeneous cohorts and should be regarded as associative rather than validated, the replication of Barreiro et al.^100^ being the principal exception. Even with these caveats, urinary vesicles occupy a strategic position in the digital urinary biopsy, because they are abundant enough for routine assay and selective enough to report defined cell types,^41^, ^44^ complementing the deeper but sparser shed‐cell pillar.

### 2.3 Pillar III: Cell‐free DNA, methylation and cell‐of‐origin mapping

The third pillar exploits a different principle, namely that dying cells release fragmented DNA that retains the epigenetic and physical signatures of its tissue of origin.^42^, ^52^ This insight was developed first in plasma along two complementary axes. The methylation axis was established when Lehmann‐Werman et al.^52^ showed that cell‐type‐specific methylation patterns in circulating DNA identify the tissue source of cell death, detecting pancreatic beta‐cell, oligodendrocyte, neuronal and other organ‐specific death in the appropriate clinical settings. Sun et al.^50^ extended this to genome‐wide methylation sequencing of plasma DNA, permitting tissue‐of‐origin mapping for prenatal, oncologic and transplant applications.

The fragmentation axis was developed in parallel: Snyder et al.^42^ showed that the fragmentation pattern of cfDNA encodes a nucleosome footprint that reports the cell types contributing to the circulating pool, Jiang et al.^112^ demonstrated that fragment length itself carries tissue‐of‐origin information, and Mouliere et al.^113^ and Cristiano et al.^114^ showed that fragment‐size selection and genome‐wide fragmentation profiles, respectively, enhance detection and classification of disease‐derived DNA. The same nucleosome‐positioning information can be pushed further toward cell state, as Ulz et al.^115^ demonstrated that nucleosome footprints at transcription start sites in plasma DNA infer the expression status of the genes in the cells of origin, which raises the prospect of reading from fragmentation alone both the identity of dying kidney cells and the transcriptional program active within them. Underlying these patterns is a definable enzymatic mechanism, since Han et al.^116^ showed that cfDNA fragmentation is generated by the coordinated action of intracellular and circulating nucleases, in particular DNASE1L3, DNASE1, and the apoptotic nuclease DFFB, which cleave preferentially in the linker regions between nucleosomes and thereby imprint the nucleosomal organization of the cell of origin onto the fragment‐end distribution. Consequently, fragmentation is not random degradation but a structured readout of chromatin state, which is what licenses its use for cell‐of‐origin inference. The conceptual advance relevant here is that methylation and fragmentomics, being cell‐type specific and stable, together allow a mixed pool of cfDNA to be decomposed into its cellular sources^48^, ^50^ (Figure 6).

**FIGURE 6:**
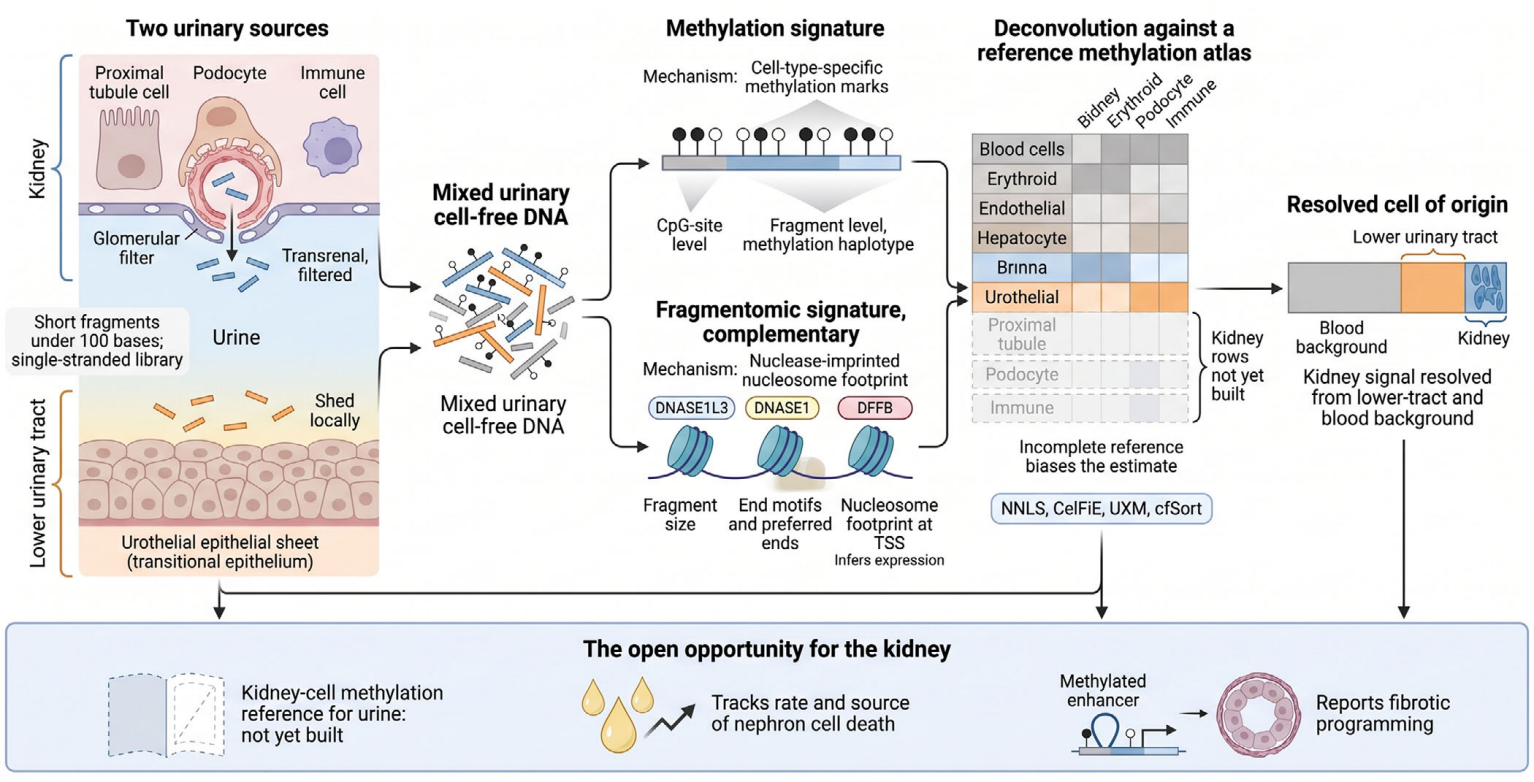
Methylation‐based cell‐of‐origin mapping of cfDNA. Conceptual illustration of how cfDNA released by dying kidney and urinary tract cells can be decomposed into its cellular sources. A mixed pool of fragments carrying cell‐type‐specific methylation marks is compared against a reference methylation atlas, and deconvolution algorithms estimate the proportional contribution of each cell type. The kidney‐derived signal, including proximal tubule, podocyte, and immune contributions, is shown being resolved against a lower urinary tract background, with fragmentomic features providing complementary information. The figure frames the unmet need for a kidney‐cell methylation reference for urinary applications. Created with BioRender: https://app.biorender.com/illustrations/6a461e33323adb311fc63115.

The decisive enabling resource has been the construction of reference methylation atlases. Moss et al.^48^ assembled a methylation atlas spanning 25 human cell types and used non‐negative least squares deconvolution to estimate the cellular composition of plasma cfDNA, showing in healthy individuals that the pool is dominated by white blood cells and erythrocyte progenitors with smaller endothelial and hepatocyte contributions, and that disease shifts these proportions in interpretable ways. Subsequently, Loyfer et al.^117^ produced a comprehensive DNA methylation atlas of normal human cell types at far greater resolution, providing the reference granularity needed to distinguish closely related cell states. Building on such references, Li et al.^118^ developed cfSort, a deep‐learning deconvolution model trained on a large tissue methylation atlas comprising 521 noncancer samples across 29 human tissues, achieving accurate tissue deconvolution for disease detection and monitoring. Complementary algorithms, including CelFiE from Caggiano et al.^119^ and MetDecode for multi‐cancer typing,^120^ have extended the statistical machinery for resolving cellular contributions to cfDNA. For the kidney specifically, methylation serves as a cell‐of‐origin tag and as a disease signal in its own right, since Ko et al.^121^ showed that cytosine methylation changes in enhancers of profibrotic genes characterize kidney fibrosis, and Gluck et al.^122^ showed that kidney methylation states improve estimation of renal function decline, which implies that a kidney‐resolved methylation readout of urinary cfDNA could report fibrotic programming as well as cellular composition.

Within nephrology, cfDNA has been validated most thoroughly in transplantation, where it provides a template for native disease applications. Bloom et al.^123^ established that donor‐derived cfDNA in plasma detects active rejection of kidney allografts, and Aubert et al.,^124^ in a large multicentre study of more than 2800 recipients, confirmed the clinical performance of cfDNA for detecting allograft rejection. Importantly, urinary cfDNA has been interrogated directly, building on the early demonstration by Botezatu et al.^54^ that DNA from cells dying within the body is excreted in urine in a transrenal manner and can be recovered for genetic analysis. Work on transrenal and urinary cfDNA, including single‐stranded library approaches that recover short and degraded fragments, has detected donor‐derived DNA and infection in transplant recipients, demonstrating that the urinary cfDNA compartment is accessible and informative.^125^, ^126^ These transplant findings establish proof of principle that cfDNA can report kidney cell death non‐invasively. They do so, however, through a genotype‐based contrast between donor and recipient that has no counterpart in native DKD, where the cells of interest share the patient's genome and must instead be separated by methylation or fragmentation.

Translating this to native DKD, however, requires solving a specific problem of cellular resolution. Urinary cfDNA is contributed substantially by lower urinary tract epithelium,^55^, ^127^ so the kidney‐derived signal, and within it the proximal tubule, podocyte, and immune contributions of interest, must be resolved against a genitourinary background. This is exactly the task that kidney‐specific methylation references, derived from the same single‐cell and spatial atlases discussed below, would enable, yet kidney‐cell‐resolved deconvolution of urinary cfDNA in DKD remains essentially unexplored. This represents one of the clearest open opportunities in the entire framework. A urinary cfDNA assay anchored to a kidney methylation reference could, in principle, quantify the rate and cellular source of nephron cell death longitudinally, complementing the transcriptional snapshots provided by the shed‐cell and vesicle pillars. Realising that possibility requires a set of analytical parameters to be established and reported rather than assumed: the kidney‐cell and lower‐urinary‐tract methylation references against which deconvolution is performed, input DNA mass, the fragment‐size distribution of the preparation, sequencing depth, the limit of detection for a kidney‐derived fraction, the normalization applied, and technical reproducibility within and between laboratories. None of these has yet been fixed for a urinary application, and whether the transrenal contribution varies with filtration barrier injury in DKD has not been tested. This pillar is therefore an opportunity rather than an established platform.

## 3 THE ANCHORING REFERENCE: SPATIAL SINGLE‐CELL ATLASES OF THE KIDNEY

The defining feature of the digital urinary biopsy is not the urinary measurement itself but the step that interprets it, namely anchoring to a spatial single‐cell reference. The value of any urinary signal depends on a map that assigns molecular features to cell states and to their positions within tissue, and over the past several years such maps have been built at increasing resolution. The first single‐cell surveys of the human kidney, including the work of Young et al.^128^ defining renal cellular identity, the chromatin and transcriptional census of the adult human kidney by Muto et al.,^129^ and the description by Stewart et al.^130^ of the spatiotemporal immune zonation of the kidney, established baseline cellular references for the healthy organ. These built on foundational single‐cell surveys of the mouse kidney by Park et al.,^131^ which nominated cellular targets of disease and demonstrated the discriminative power of the approach. These healthy‐organ references were soon extended into disease. For DKD specifically, Wilson et al.^132^ provided the first single‐nucleus transcriptomic portrait of early human disease, profiling more than 23 000 nuclei and identifying altered potassium‐secretory programs and early injury signatures. The same group, in work led by Wilson and Muto,^133^ then combined single‐cell transcriptomics with chromatin accessibility and showed expansion of a pro‐inflammatory injured proximal tubule state marked by vascular cell adhesion molecule 1, enriched for nuclear factor kappa B signalling, with genetic fine‐mapping linking these cells to CKD and filtration heritability. This injured proximal tubule state, recurring across kidney diseases and conserved between species, is a strong candidate anchor for urinary signals, because the same cells are recovered in urine sediment and reported by vesicle cargo.^49^, ^69^ The conservation of failed‐repair tubular states was further established by Kirita et al.^67^ in experimental injury and by Gerhardt et al.,^68^ who defined persistent injured‐cell states in the transition from acute injury to chronic disease. Complementing the tubular focus, single‐cell profiling of glomerular cells by Fu et al.^104^ defined dynamic podocyte and endothelial changes in experimental diabetic disease, and Menon et al.^134^ identified an endothelial marker of remission in glomerular disease, extending the cellular reference to the glomerular compartment. Balzer et al.^135^ further distinguished druggable pathways underlying adaptive versus fibrotic regeneration, while comprehensive profiling by Li et al.^136^ defined shared and unique epithelial injury responses during fibrosis, refining the cell‐state vocabulary against which urinary signals would be matched. The reference that now serves as the field benchmark is the integrated atlas of the Kidney Precision Medicine Project and its collaborators, whose rationale and design established a framework for linking molecular phenotype to clinical outcome and whose reference tissue atlas provided a transcriptomic baseline for the human kidney.^137^, ^138^ Lake et al.^139^ assembled a multimodal atlas of the healthy and injured human kidney, combining single‐cell and single‐nucleus transcriptomics, chromatin accessibility, and spatial imaging to define more than 50 cell types and states, including degenerative and adaptive states organized into injury neighbourhoods that associate with clinical outcomes.

These atlases were enabled by a succession of spatial‐profiling technologies whose lineage merits explicit credit. Sequencing‐based spatial transcriptomics, introduced by Ståhl et al.^140^ and advanced to near‐cellular resolution by the Slide‐seq methods of Rodriques et al.^141^ and Stickels et al.,^142^ placed transcriptomes in tissue coordinates, while imaging‐based approaches, including the multiplexed single‐molecule method of Chen et al.^143^ and the highly multiplexed protein imaging of Goltsev et al.,^144^ resolved individual transcripts and proteins in situ. The convergence of these modalities into integrated single‐cell and spatial multi‐omics, surveyed by Vandereyken et al.,^145^ together with the recognition of spatially resolved transcriptomics as a method of the year^146^ and the deployment of commercial imaging platforms that resolve single transcripts within intact tissue,^147^ supplied the resolution required to define disease‐relevant niches. Building on that resolution, integration of spatial and single‐cell data, demonstrated by Melo Ferreira et al.^148^ and by spatially resolved analyses of injury,^149^ localizes epithelial‐immune cross‐talk within tissue architecture, resolving the interactions rather than the cell types alone. Importantly, divergence between mouse and human kidney gene expression, documented by Cheval et al.,^150^ cautions against over‐reliance on animal references for anchoring human urinary signals.

Building towards disease mechanism, Abedini, Levinsohn, et al.^151^ integrated more than half a million single cells and nuclei with spatial data from healthy, diabetic, and hypertensive kidneys and defined fibrotic microenvironments nucleated by injured proximal tubule cells, showing that the abundance of these microenvironments predicts progression of CKD (Figure 7). This is conceptually decisive for the present framework, because it demonstrates that specific, spatially organized cell‐state niches carry prognostic information, which is the property a urinary readout would aim to recover.

**FIGURE 7:**
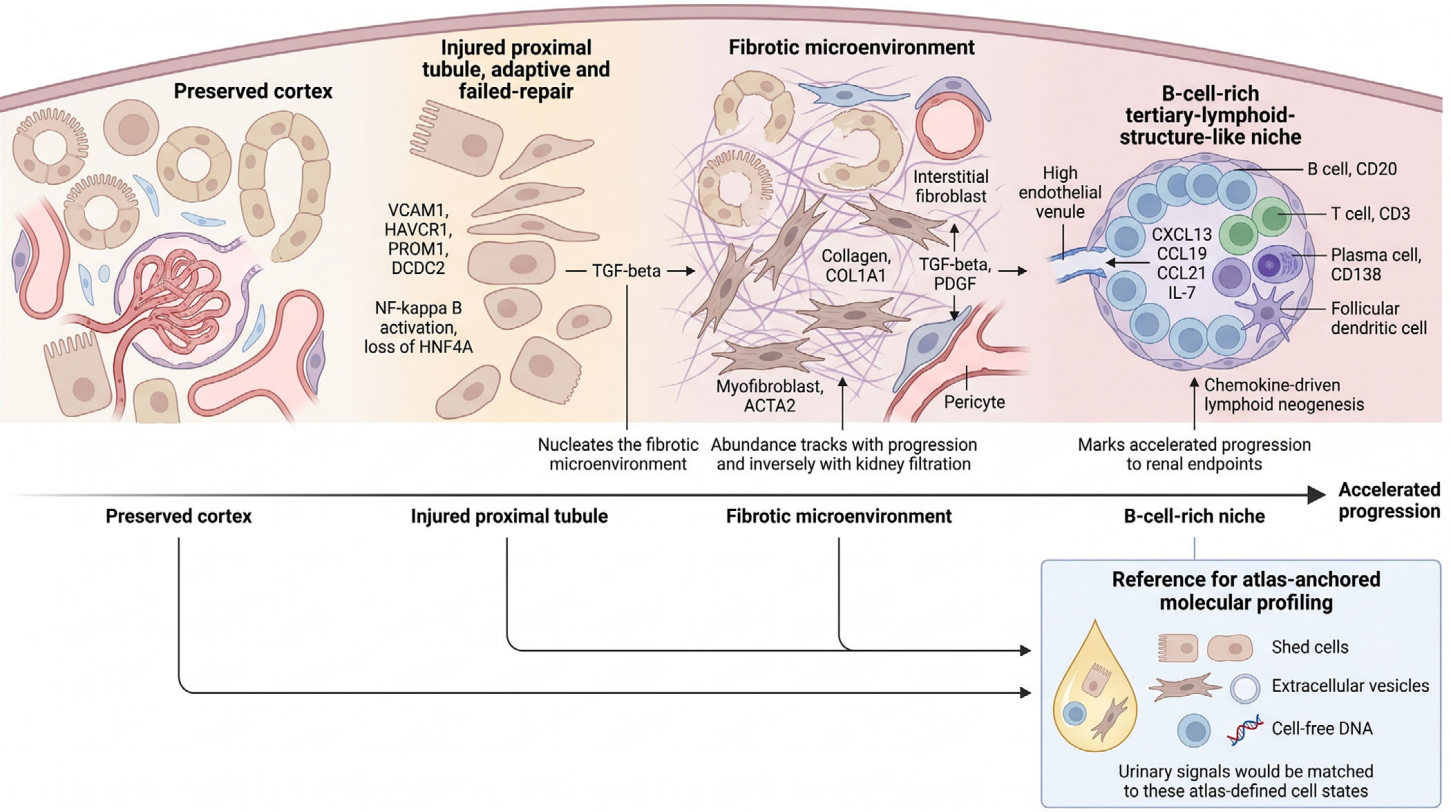
Spatial cell states and microenvironments of the diabetic kidney. Spatially organized depiction of the disease‐relevant niches defined by single‐cell and spatial atlases. The injured proximal tubule state, the fibrotic microenvironment nucleated by injured tubular cells, and the B‐cell‐rich tertiary‐lymphoid‐structure‐like immune niche within the profibrotic tubular compartment are positioned within representative tissue architecture. Associations with progression to renal endpoints are indicated. The figure presents the reference cell states and niches to which urinary signals would be anchored for atlas‐anchored molecular profiling. It is a schematic synthesis of published atlas findings and does not display primary or public data. Created with BioRender: https://app.biorender.com/illustrations/6ab3886d5deacbc5cfd0102b.

Most recently, the spatial resolution of DKD has advanced further. Dumoulin et al.^152^ constructed a cross‐platform spatial atlas of DKD using imaging‐based spatial transcriptomics together with single‐nucleus sequencing and identified a B‐cell‐rich immune microenvironment, resembling a tertiary lymphoid structure, embedded within the profibrotic tubular niche, which defined a patient subset with accelerated progression to renal endpoints. Notably, the same study derived tissue biomarkers and a matched circulating protein panel that stratified patients in a population biobank and improved prediction beyond clinical models.^152^ This finding is important to the present argument in two ways. It confirms that spatially defined immune niches mark a clinically meaningful endotype, and it sets the most competitive contemporary benchmark, because it achieves stratification using a plasma panel anchored to a spatial atlas. The differentiation of the digital urinary biopsy must therefore be explicit. Where the plasma panel reports a niche indirectly through circulating proteins, a urinary, cell‐resolved readout could in principle detect the constituent cells, vesicles, and cfDNA of that niche directly and longitudinally, including the shed B and plasma cells, the injured tubular cells, and the fibrosis‐associated signals that compose it.^76^ The niche itself has a definable developmental mechanism, since ectopic lymphoid structures of this kind arise through chemokine‐driven neogenesis, in which local expression of CXCL13 and the CCL19 and CCL21 axis, together with lymphotoxin signaling and the induction of high endothelial venules, organizes recruited B and T cells into follicle‐like aggregates, as reviewed by Pitzalis et al.^153^ Detecting the shed cellular and molecular constituents of such a structure in urine would therefore report an identifiable immunopathological program rather than a nonspecific inflammatory signal. Correspondingly, the fibrotic compartment that surrounds it has a traceable cellular origin, because Humphreys et al.^74^ established by genetic fate tracing that myofibroblasts derive predominantly from resident pericytes and interstitial fibroblasts rather than from epithelial transdifferentiation, while TGF‐β signaling, as noted earlier, activates these progenitors into the matrix‐secreting state.^21^ Fibrosis‐niche biology, further defined by Kuppe et al.^75^ in their decoding of myofibroblast origins in human kidney fibrosis, supplies the additional reference states such a readout would target. Altogether, the atlases now provide a sufficiently detailed and outcome‐linked reference to make anchoring of urinary signals a tractable goal.

## 4 ATLAS‐ANCHORED MOLECULAR PROFILING AS AN INVERSE PROBLEM: DECONVOLUTION, REFERENCE MAPPING AND INTEGRATION

The inference of kidney cell states from a urinary measurement is, in formal terms, an inverse problem. A urinary profile can be modelled as a mixture in which the observed signal equals a reference matrix of cell‐state‐specific features multiplied by an unknown vector of cell‐state contributions, plus noise and contamination from the lower urinary tract. Because the three compartments differ in what is released, how release is selected, and how it is measured, this mixture must be written once for each compartment rather than once for the framework. Each requires its own reference matrix and its own sampling function: the spatial transcriptomic atlas for shed cells, whose recovery is biased toward lymphocytes and against degraded epithelium;^43^, ^49^ a cargo‐specific signature matrix for vesicles, since sorting makes cargo non‐proportional to parent‐cell transcript abundance;^51^ and a kidney‐cell methylation atlas for cfDNA, a different object from the transcriptomic atlas that does not yet exist at the required resolution,^117^, ^122^ read against a fragmentation regime particular to urine, where the modal fragment is near 81 base pairs and fragmentation tracks urinary tract epithelium more closely than plasma does.^127^ Every compartment also needs two background terms, one for the lower urinary tract and one for hematopoietic cells.^48^, ^125^ Finally, the compartments must be partitioned preanalytically rather than assumed independent, because extracellular DNA co‐isolates with vesicle preparations without being packaged inside exosomes,^154^ so the same molecules could otherwise be counted as both vesicular cargo and cfDNA. Within each of these compartment‐specific models, recovering the contribution vector from the observation is mathematically ill‐posed, because many compositions can produce a similar mixed signal, because the reference matrix is itself estimated with error, and because urinary sampling is sparse and biased.^155^, ^156^ Stable solution therefore requires regularization and an informative prior, and the compartment‐matched atlas supplies that prior by constraining which cell states exist, what features distinguish them, and how they are spatially organized in the reference tissue. Viewed in this way, the three computational operations described below, namely deconvolution, reference mapping, and multi‐omic integration, are not a loose toolkit but successive components of a single regularized inversion that maps biofluid observations onto atlas‐defined cell states.

Given a urinary signal and a spatial reference atlas, the first operation is deconvolution, the estimation of cell‐state composition from a mixed measurement. The methods have matured rapidly, initially for bulk tissue. Newman et al.^157^, ^158^ introduced CIBERSORT and its successor CIBERSORTx, which infer cell‐type proportions and cell‐type‐specific expression from bulk profiles using a signature matrix derived from single‐cell references. Subsequently, a second generation built single‐cell‐informed estimators, including the multi‐subject reference method MuSiC of Wang et al.,^159^ the robust integration approach BisqueRNA of Jew et al.,^160^ the deep‐learning estimator Scaden of Menden et al.,^161^ and the Bayesian method BayesPrism of Chu et al.,^162^ each designed to propagate single‐cell reference information into mixed measurements while controlling for cross‐sample and cross‐platform variation. For spatial data, a family of probabilistic and deep‐learning methods has emerged, including cell2location from Kleshchevnikov et al.,^163^ Tangram from Biancalani et al.,^164^ robust cell‐type decomposition from Cable et al.,^165^ and the conditional autoregressive method CARD from Ma and Zhou.^166^ Across these families, systematic benchmarking of spatial deconvolution pipelines has generally placed cell2location, CARD, and Tangram among the strongest performers for mapping cell states to tissue positions,^156^ while broader deconvolution benchmarks by Avila Cobos et al.^155^ show that accuracy depends heavily on reference quality, signature‐gene selection, and the degree of mismatch between reference and query, which is the central practical risk when a solid‐tissue atlas is used to interpret a urinary signal.

That risk is specific rather than general. Spatial mapping methods such as cell2location and Tangram were built to assign cells to positions within a tissue section and assume a query measured on intact tissue,^163^, ^164^ while signature‐matrix methods of the CIBERSORT family assume that mixture and reference are measured on comparable platforms.^157^, ^158^ Neither assumption holds in urine, where shed cells are degraded, vesicle cargo is selectively loaded rather than proportional, and cfDNA is read as methylation rather than as expression. These methods are therefore starting points that require adaptation and benchmarking in the urinary setting, not tools that transfer unchanged. The conceptual pipeline by which these tools convert urinary signals into an inferred cell‐state profile is shown in Figure 8.

**FIGURE 8:**
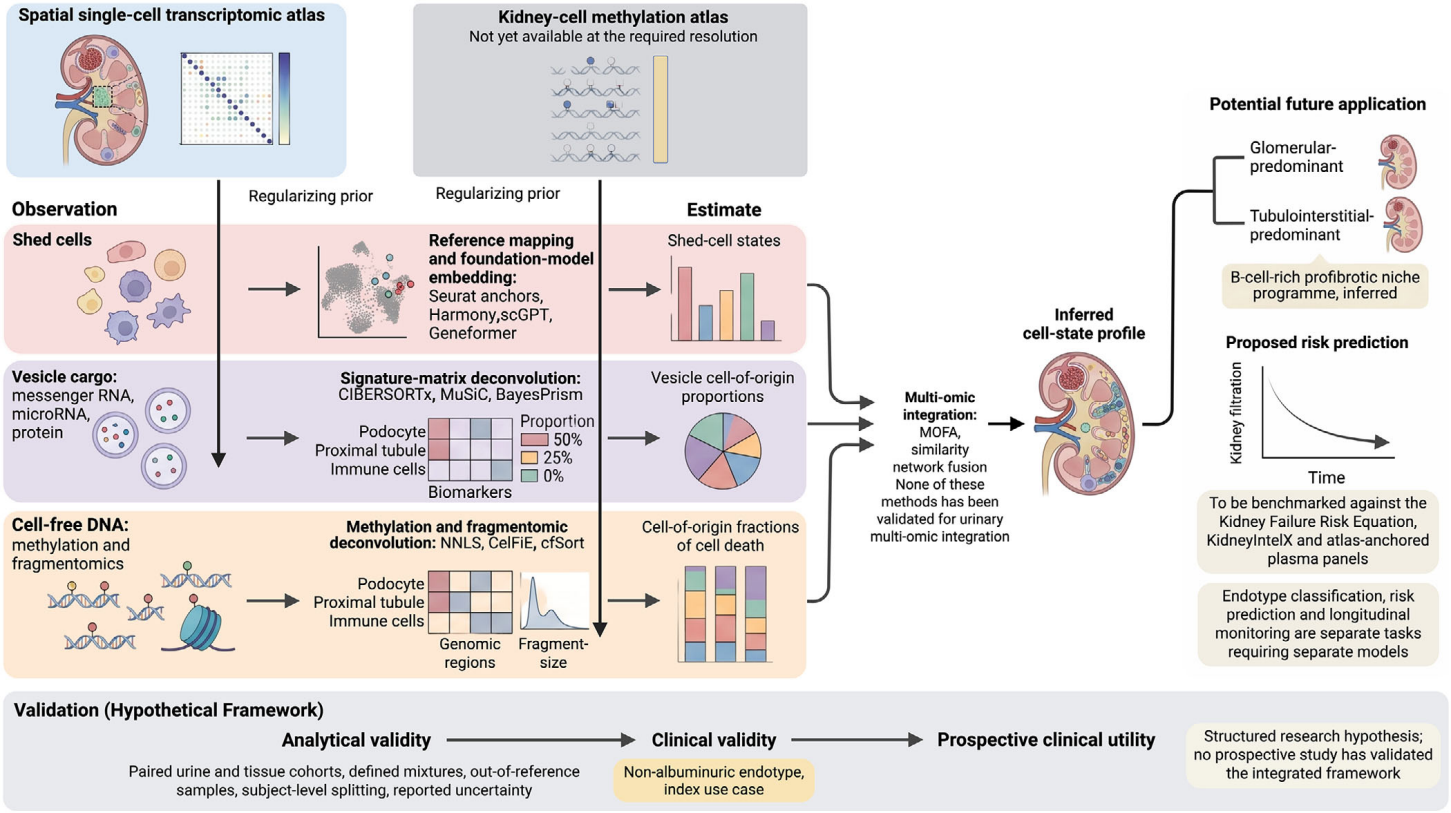
Atlas‐anchored molecular profiling as an inverse problem: the analytical pipeline and potential future application. Integrated computational workflow framed as a regularized inversion. Per‐compartment urinary signals are processed by matched methods (reference mapping and foundation‐model embedding for shed cells, signature‐matrix deconvolution for vesicle cargo, and methylation and fragmentomic deconvolution for cfDNA), each constrained by a compartment‐matched atlas prior, namely the spatial single‐cell transcriptomic atlas for shed cells and vesicle cargo and a separate kidney‐cell methylation atlas, not yet available at the required resolution, for cfDNA, then fused by multi‐omic integration into a single inferred cell‐state profile that solves the otherwise ill‐posed mapping from biofluid observation to kidney cell composition. This profile is intended to drive endotype classification and risk prediction, which would be benchmarked against established tools including the KFRE, KidneyIntelX, and atlas‐anchored plasma panels. The pipeline is a proposal, and none of the named methods has yet been validated for urinary multi‐omic integration. A staged validation roadmap, progressing from analytical validity through clinical validity to prospective demonstration of clinical utility, is shown as the translational pathway, with the non‐albuminuric endotype highlighted as the index use case. Created with BioRender: https://app.biorender.com/illustrations/6ab38b9e56d45129334de617.

Reference mapping, as distinct from proportion estimation, supplies the second computational ingredient. Methods that transfer labels from an annotated reference to a query dataset, including the anchor‐based integration developed by Stuart et al.^167^ and refined by Hao et al.,^168^ together with the batch‐correction method Harmony of Korsunsky et al.,^169^ allow urinary cells of uncertain identity to be assigned to atlas‐defined states while controlling for technical variation between the reference tissue and the urinary query, a problem reviewed comprehensively by Stuart and Satija.^170^ This is essential because urine and solid kidney differ systematically in dissociation, ambient RNA, and cell viability, so naive comparison would conflate biological and technical differences. More recently, single‐cell foundation models have offered a route to robust embedding and transfer. scGPT, developed by Cui et al.^171^ and pretrained on tens of millions of cells, and Geneformer, developed by Theodoris et al.^172^ through transfer learning on large transcriptomic corpora, generate representations that support cell‐type annotation and cross‐dataset mapping. Subsequent models, including the large‐scale scFoundation of Hao et al.^173^ and the universal cell embeddings of Rosen et al.,^174^ extend this paradigm toward representations intended to generalize across tissues and platforms, and the broader programme of building a virtual cell, articulated by Bunne et al.,^175^ frames such models as components of a predictive simulation of cellular state. In the present context, these representations could in principle stabilize the assignment of sparse, noisy urinary cells to atlas states by embedding query and reference in a shared latent space, although their behaviour on degraded biofluid material has not been characterized. Multi‐omic integration methods then fuse the pillars. Multi‐omics factor analysis, introduced by Argelaguet et al.^176^ and extended in its multimodal single‐cell form,^177^ together with similarity network fusion from Wang et al.,^178^ provide statistical frameworks for combining transcriptomic, vesicular, and epigenetic readouts into joint patient representations, and Miao, Humphreys, et al.^179^ have reviewed how such integration applies specifically to kidney single‐cell data. Bringing these elements together defines what we term atlas‐anchored molecular profiling: the estimation, from urinary measurements, of which kidney cell states are present, in what proportion, and whether niche‐associated molecular programs are active. Four inferences must be separated, since each rests on a different method and assumption. Cell‐of‐origin inference assigns a urinary signal to a parent cell type, as methylation deconvolution does for cfDNA;^48^, ^52^ cell‐state identification assigns it to a transcriptional or epigenetic state within that type, which is the task of reference mapping;^167^, ^168^ abundance estimation quantifies each state's contribution to the mixture, the task of signature‐matrix and single‐cell‐informed deconvolution;^157^, ^159^ and spatial niche reconstruction would recover the physical arrangement of cells in tissue, which is what cell2location and Tangram perform on intact sections.^163^, ^164^ Only the first three are accessible from urine, though not because the query lacks spatial coordinates. Position can be reconstructed from dissociated cells that carry none: by matching each cell against a positional reference atlas, which in one study assigned cells to precise locations with a success rate of 81%;^180^, ^181^ by using landmark genes taken from microdissected tissue, which resolved zonation along the intestinal villus;^182^ or by exploiting the correspondence between transcriptional similarity and physical proximity, which has been applied de novo to whole kidney among other tissues.^183^ Two constraints nonetheless apply. The accuracy of such methods depends heavily on context, dataset size and platform,^184^ and each assumes a population that covers the tissue, which urine does not supply, since shedding is biased toward detached and dying cells and the recovered population is pooled over time and admixed with lower urinary tract material.^43^, ^49^ The limit is therefore one of sampling rather than of information in principle, and on present sampling no urinary measurement recovers the architecture of the patient sampled; the atlas contributes spatial information only by recording where a given state resides in reference tissue. Ma et al.^69^ illustrate both the reach and the limit of this operation, since mapping urinary single‐cell profiles onto a spatial diabetic kidney atlas placed fibrosis‐committed and proliferative adaptive tubular cells near the glomerular region of the reference, which locates a state within reference tissue rather than reconstructing that patient's architecture. Accordingly, we retain the phrase virtual histology only as informal shorthand for this bounded operation, and any inference at the level of a niche will require paired urine and tissue sampling from the same patients, spatial validation in the tissue arm of the kind reported for fibrotic and immune microenvironments,^151^, ^152^ and evidence that several urinary signals co‐vary in a manner specific to that niche.

The logic proceeds in stages, each drawing on a method class established in an adjacent setting. Shed‐cell transcriptomes are mapped to atlas‐defined states by reference transfer;^167^, ^168^ vesicular RNA and protein cargo are deconvolved to cell‐of‐origin proportions using signature matrices built from the same atlases;^157^, ^158^ and urinary cfDNA is decomposed by methylation deconvolution against a kidney‐cell methylation reference.^48^, ^117^ The resulting per‐compartment estimates are then integrated by multi‐omic factor analysis or similarity network fusion^176^, ^178^ into a single cell‐state profile that reports, non‐invasively, which cell states are active and in what proportion, rather than what a biopsy would show. Such a profile would support endotype assignment, for instance distinguishing a glomerular‐predominant from a tubulointerstitial‐predominant process, or detecting the molecular program associated with the B‐cell‐rich profibrotic niche that marks accelerated progression.^152^ Finally, the cell‐state profile feeds risk models. Established statistical learning methods, including penalized regression and random forests of the kind used in KidneyIntelX, together with foundation‐model embeddings, can map the inferred profile to predicted filtration trajectory or kidney failure risk.^36^, ^171^ The advantage over a scalar biomarker is interpretability; because the prediction is grounded in identified cell states, it can in principle indicate which biological process is driving a patient's risk, and not merely that the risk exists, thereby supporting mechanism‐matched therapy.

Three requirements follow for any such model. Methods must be benchmarked against simple baselines on defined mixtures and on paired urine and tissue specimens, with out‐of‐reference samples included and uncertainty reported,^155^, ^156^ rather than assumed to transfer from tissue applications. Data must be split at the level of the subject and not the cell, with nested cross‐validation, external validation, and explicit control for leakage through shared atlas references or batch structure.^185^ And the three tasks addressed here, namely risk prediction, endotype classification, and longitudinal monitoring of treatment response, differ in endpoint and time horizon and require separate models and separate evidence rather than a single one.

## 5 THREATS TO VALIDITY AND THE LIMITS OF RESOLUTION

The framework must confront the physical limits of the measurement before any claim of clinical readiness. Urinary cell yields are low and highly variable, so that a single specimen may contain too few cells of a given state to estimate its abundance with confidence, and the detection of a rare but informative population, for example a profibrotic adaptive tubular cell present at a few percent, is bounded by sampling noise in the same way that any count‐based assay is bounded.^43^, ^49^ The cfDNA compartment faces an analogous floor, because the kidney‐derived fraction is small relative to contributions from the lower urinary tract and from systemic cell death,^55^, ^127^ which sets a limit of detection for kidney‐specific signal that depends on sequencing depth and on the resolution of the methylation reference. Recognizing these floors reframes the engineering target. The objective is to measure the three compartments deeply enough, and to pool information across them, so that the integrated estimate clears the noise threshold for the cell states that carry prognostic weight. A second and more fundamental concern is representativeness. The inference that a urinary cell‐state profile mirrors the parenchyma assumes that cells enter urine in proportion to their tissue abundance, yet shedding is a biased process. Dying, detaching, and senescent cells are released preferentially, viable resident cells of some lineages are retained, and degradation during urinary transit further distorts the recovered population, with lymphocytes surviving better than fragile epithelial and urothelial cells.^43^, ^49^ The same selection operates, in different form, on vesicles and cfDNA, whose release reflects secretion and cell death rather than steady‐state composition.^51^, ^116^ Consequently, the three pillars do not report tissue composition directly but report it through compartment‐specific sampling functions, and recovering the underlying tissue state requires modelling those functions rather than assuming they are uniform. Treating the sampling bias as a parameter to be estimated, rather than as noise to be ignored, is a prerequisite for valid anchoring.

Confounding from outside the kidney compounds the problem. Urinary cfDNA is contributed substantially by urothelium, and its origin and fragmentation differ from those of plasma, as Cheng et al.^55^ showed in characterizing the urinary cfDNA pool. Haematuria introduces blood‐derived cells and DNA, urinary tract infection perturbs the immune signal, and anatomical sex shapes the background through prostatic and genital contributions, while menstrual contamination affects female specimens.^125^, ^126^ Each of these can masquerade as, or obscure, a kidney signal. Robust anchoring therefore depends on explicit deconvolution of non‐kidney sources, on quality‐control gates that flag contaminated specimens, and on reference panels that include the relevant lower urinary tract and hematopoietic cell states so that they are partitioned out rather than misassigned. The three pillars also differ in the time window they sample, which complicates their integration. cfDNA reflects cell death over a short and recent interval set by its rapid clearance,^52^ shed cells capture the transcriptional state of recently detached cells,^43^, ^49^ and vesicles integrate secretion over a longer and less defined period,^46^, ^92^ so that a single specimen superimposes signals from distinct temporal scales that must be aligned rather than averaged.

Beyond timing lies a deeper epistemic obstacle, namely the ground‐truth paradox, since validating an atlas‐anchored profile appears to require the tissue diagnosis it is meant to replace. Several strategies dissolve this circularity without demanding routine biopsy. Paired urine and tissue cohorts, of the kind assembled by the Kidney Precision Medicine Project,^137^, ^138^ provide matched references for a finite training set; in silico mixtures built from atlas single‐cell profiles create synthetic urinary signals of known composition for benchmarking deconvolution, as published benchmarks of these methods have done;^155^, ^156^ allograft recipients supply an internal ground truth, because donor‐recipient genotype mismatch independently quantifies the kidney‐derived fraction;^123^, ^125^ and controlled shedding models in organoids and xenografts could allow the sampling functions to be measured directly. The staged validation pathway that follows from these considerations is summarized in Figure 8. Only once the measurement floors, sampling biases, and confounders are characterized can the anchored estimate be trusted as a faithful inference rather than a merely plausible one.

## 6 FROM CONCEPT TO CLINIC: TRANSLATIONAL ROADMAP AND CHALLENGES

The path from this framework to clinical use runs through standardization, validation, demonstrated incremental value, and affordability, and each presents defined obstacles. The first is preanalytical standardization. Urinary cell yield, vesicle integrity, and cfDNA recovery are all sensitive to collection method, time to processing, temperature, and stabilization,^43^, ^49^, ^186^ and without harmonized protocols, cross‐site comparison and reference‐atlas anchoring will fail. The reporting frameworks already established for EVs by the International Society for Extracellular Vesicles provide a template, and analogous standards for urinary cell and cfDNA collection will be required.^101^, ^108^ These standards cannot simply be merged, because the three compartments impose conflicting demands on one specimen. Shed cells require prompt processing of fresh urine, since viability and transcriptional integrity decline with transit and storage.^43^, ^49^ Vesicles require protease inhibitors and storage at ‐80°C with vigorous resuspension after thawing, since freezing at ‐20°C causes major loss of recovery whereas recovery after ‐80°C is almost complete,^186^ and particle counts shift further with hydration state.^106^ cfDNA is the most tolerant compartment, its urinary fragmentation patterns being robust to home collection and to delays in processing,^127^ yet the same delay lyses cells and releases genomic DNA that contaminates it. No single handling protocol optimises all three. A digital urinary biopsy therefore requires the specimen to be divided into compartment‐specific aliquots at collection, and that division, rather than any one downstream assay, becomes the first object of standardization. Furthermore, the reference atlases must be extended to include the molecular layers that urinary anchoring requires, in particular kidney‐cell methylation references for cfDNA deconvolution, which do not yet exist at the needed resolution despite the availability of the normal methylation atlas of Loyfer et al.^117^

The second obstacle is analytical and clinical validation, for which precedent exists. KidneyIntelX progressed through analytical validation of its assays and prospective evaluation, including in the CANVAS trial population, and in June 2023 received De Novo marketing authorization, which created a new class II classification for prognostic tests assessing CKD progression, providing a concrete pathway that an anchored urinary assay could follow.^36^, ^187^, ^188^ That precedent is bounded. The authorized indication is an aid to assessing the risk of a sustained decline in filtration of at least 40% within 5 years, in adults with T2D and either an eGFR of 30 to 59 mL/min/1.73 m^2^ or a rate of 60 or above with an albumin‐to‐creatinine ratio of at least 30 mg/g, and the record states that the test is not intended for serial monitoring of disease progression or for monitoring the effect of any therapeutic product.^188^ The indication therefore does not extend to the normoalbuminuric population with preserved filtration targeted here, and the longitudinal monitoring proposed in this framework would require its own evidence of clinical utility rather than inheriting this precedent.

The evidence base beneath such a pathway is uneven. Feasibility is established for urinary single‐cell sequencing and vesicular messenger RNA profiling,^43^, ^93^ and associative performance is established for several tubular markers, since urinary KIM‐1, monocyte chemoattractant protein‐1, and the epidermal growth factor to monocyte chemoattractant protein‐1 ratio predict progression in diabetes,^57^, ^60^ with independent cohorts reinforcing these associations and cataloguing the standards required to translate biomarkers into prognostic tools.^56^, ^185^, ^189^, ^190^ The integration of such markers into molecular classifications, advanced through multi‐omic and precision‐nephrology efforts that resolved tumour necrosis factor activation variability across glomerular diseases, points toward the endotype‐level stratification the digital urinary biopsy seeks.^191^, ^192^ What remains entirely unproven is clinical utility of the integrated, atlas‐anchored framework, which no prospective study has evaluated; the framework is, at present, a structured hypothesis supported by feasibility, not a validated test.

The third obstacle is demonstration of incremental value against strong incumbents, and here the benchmarks are demanding. Any digital urinary biopsy must improve on albuminuria and eGFR, on the KFRE and on multimarker tools such as KidneyIntelX.^36^, ^37^, ^38^ These are not universal benchmarks, and comparison must be made within the population, endpoint and time horizon for which each was derived. The KFRE, for example, was developed in referred patients with CKD stages 3 to 5 and an eGFR of 10–59 mL/min/1.73 m^2^, where its concordance statistic was 0.917 in the development cohort and 0.841 on external validation;^37^ it therefore does not apply to normoalbuminuric patients with preserved filtration, who are the population this framework targets. The AUC near 0.77 reported for KidneyIntelX is likewise specific to its cohort and endpoint.^36^ More pointedly, it must justify its added complexity against the plasma protein panel of Dumoulin et al.,^152^ which already stratifies a spatially defined DKD endotype from blood. That study is a tissue‐derived comparator rather than a validation of urinary anchoring: the atlas was built from kidney tissue using imaging‐based spatial transcriptomics with single‐nucleus sequencing, and the matched protein panel was evaluated in the circulation of a population biobank, not in urine.^152^

The argument for urine rests on three claims that require empirical support. First, that a urinary, cell‐resolved readout captures the non‐albuminuric and rapidly progressive phenotypes that current markers do not resolve.^12^, ^14^ Second, that anchoring to spatial atlases reports the molecular programs associated with disease‐relevant niches, such as the B‐cell‐rich profibrotic microenvironment,^152^ that scalar markers cannot. Third, that the longitudinal accessibility of urine enables monitoring of treatment response at the level of cell state, for which vesicle proteomics offers the nearest precedent.^102^ A study that demonstrated prediction of filtration slope or kidney failure materially exceeding these incumbents, with specific capture of non‐albuminuric endotypes, would justify advancement to multicentre validation. Conversely, failure to exceed a plasma panel would indicate that the additional effort of urinary cell capture is unwarranted, and this falsifiable framing is a strength rather than a weakness of the concept.

The fourth obstacle is cost, and it constrains the form any deployment could take. The incumbent against which this framework would compete is a urine albumin measurement, and even that is not unconditionally economical. Indeed, a Markov cohort analysis of population‐wide screening found one‐time screening for albuminuria at age 55 years to cost $86 300 per quality‐adjusted life‐year gained, with screening every ten years to age 75 remaining below $100 000 per quality‐adjusted life‐year; when the assumed benefit of SGLT2 inhibition was reduced by 30%, those figures rose to between $145 400 and $182 600, at which point price reductions would be required for screening to remain cost‐effective.^193^ Against that benchmark, three specialized assays performed annually across the diabetic population could not meet such a threshold. The resolution, if there is one, lies in separating discovery from deployment. Specifically, single‐cell sequencing, vesicle multi‐omics and methylation deconvolution are discovery instruments whose product is a compact feature set, and the deployed test would be a targeted assay reading those features. Suthanthiran et al.^65^ demonstrated the pattern when a three‐gene urinary signature was measured by quantitative polymerase chain reaction across 4300 specimens from 485 patients in a multicentre study, and the three‐gene panel nominated from urinary single‐cell data illustrates the same logic in diabetes.^84^ A tiered deployment follows: albuminuria and filtration screening first, with an anchored urinary assay reserved for patients already flagged or for endotype assignment where it would change management. Should no compact panel reproduce the multi‐compartment signal, the framework would remain a discovery tool rather than a clinical test.

Underlying the first three obstacles is the formal framework of biomarker qualification, which separates analytical validity, clinical validity and clinical utility.^185^ Analytical validity requires that urinary cell, vesicle, and cfDNA measurements be accurate, reproducible, and robust to preanalytical variation against the reporting standards already defined for vesicles,^101^, ^108^ a bar that current single‐centre studies do not yet meet. Meeting it means reporting, for each compartment separately, technical replicates, within‐run and between‐run precision, recovery, analyte stability across storage and freeze‐thaw, limits of detection and of quantification, batch structure and its correction, and reproducibility across centres. Reproducibility at the level of the model requires in addition that feature lists, reference matrices, training and test datasets, model versions and code accompany any implementation. Clinical validity requires that the anchored cell‐state estimate associate reproducibly with a clinical state or outcome across independent cohorts. Clinical utility, the highest and least‐met standard, requires evidence that acting on the estimate improves outcomes relative to existing care. Mapping each pillar onto this framework clarifies where effort is needed, since feasibility addresses only the first stage.

The non‐albuminuric phenotype offers a concrete test case that runs through all three pillars. In such a patient, the shed‐cell pillar would be expected to recover injured and adaptive proximal tubule states in the absence of a dominant podocyte injury signature;^49^, ^69^ the vesicle pillar would report a proximal tubule stress cargo without a commensurate rise in glomerular markers;^93^ and the cfDNA pillar would register tubular and interstitial cell death against a stable glomerular background.^48^, ^117^ A coherent tubulointerstitial signal across the three compartments, in a patient whose albumin excretion is normal, is a pattern that routine measurement of albumin excretion and filtration rate cannot produce, because those tests report function rather than the process generating it. This is set out as a hypothesis to be tested, not as an established biological definition of the phenotype.

A further argument for urine, distinct from single‐time‐point classification, is longitudinal monitoring of treatment response at the level of cell state. Disease‐modifying therapies now act on identifiable cellular programs. For instance, SGLT2 inhibition, shown in the CREDENCE trial by Perkovic et al.^194^ to reduce kidney failure in diabetic nephropathy, relieves proximal tubule workload, whereas mineralocorticoid receptor antagonism targets inflammatory and fibrotic programs.^6^ Accordingly, a readout that tracks the abundance of injured proximal tubule or profibrotic states over time could, in principle, report whether a given therapy is engaging its cellular target in an individual patient, a capability no static risk score provides. Urinary vesicle proteomics has already produced drug‐specific signatures in a randomized comparison of four agents,^102^ which is the nearest existing evidence that such monitoring is attainable, although it reports protein abundance rather than cell state. Complementary molecular layers strengthen this prospect, since urinary metabolomic signatures of mitochondrial dysfunction, defined by Sharma et al.,^195^ associate with DKD and could be integrated with the cell‐state readout to capture metabolic as well as transcriptional dimensions of tubular stress. It is repeated and non‐invasive sampling that makes urine, rather than tissue, the natural substrate for this monitoring role.

Several further directions warrant emphasis. Integration across the three pillars is likely to outperform any single compartment, because shed cells, vesicles, and cfDNA report overlapping but distinct aspects of the same disease, and multi‐omic fusion methods are well suited to combining them.^176^, ^179^ Continued advances in spatial multi‐omic technologies, including spatial metabolomics, will refine the reference against which urinary signals are interpreted, improving the granularity of atlas‐anchored molecular profiling over time.^139^, ^152^

Equity considerations are central, because reference atlases skewed toward particular ancestries will produce biased anchoring, so deliberate inclusion of diverse populations in atlas construction is a prerequisite for valid application across the global diabetic population that bears most of the disease burden.^2^, ^4^ Ancestry is not the only axis. Age, sex, diabetes type and the aetiology of coexisting kidney disease each alter cell‐state composition and the relationship between a urinary signal and an outcome,^23^, ^107^ so calibration must be demonstrated within subgroups, for the atlas and for the clinical model, rather than inferred from aggregate performance. Precedents in genomics and in nephrology are instructive. European ancestry dominates genetic association studies, which limits the transfer of risk prediction across populations;^196^ a widely deployed clinical risk algorithm was found to under‐refer Black patients because it predicted health‐care cost rather than illness, and correcting it would have raised the proportion of Black patients receiving additional help from 17.7% to 46.5%;^197^ and the eGFR itself was refitted without race once race was recognised as a social rather than a biological construct.^198^ A cell‐state readout built on skewed references and evaluated only in aggregate would repeat these errors.

The data also raise governance questions that a urinary assay inherits rather than avoids. Urinary cfDNA and shed cells carry the patient's germline sequence,^54^, ^125^ so this framework generates re‐identifiable high‐dimensional data from a specimen patients may regard as anonymous waste. Consent must therefore cover secondary use and deposition into reference atlases explicitly, governance must specify who holds raw sequence and for how long, and a policy is required for incidental findings, since such assays can reveal germline variants, malignancy of the urinary tract, and infection that lie outside the question asked. These provisions belong in study design from the outset rather than being added afterwards.

## 7 CONCLUSION

Overall, the digital urinary biopsy reframes a familiar biofluid as a structured, cell‐resolved window onto the diabetic kidney. Each of the three urinary pillars has feasibility evidence that it carries renal information, although none has been validated as a clinical assay in DKD; the spatial transcriptomic atlases that would anchor the cellular and vesicular compartments now exist and are linked to outcomes, although the kidney‐cell methylation atlas required for the cfDNA compartment does not yet exist at the necessary resolution; and the computational methods needed to connect signal to reference are mature for adjacent applications, although none has been benchmarked on urinary material. The central conceptual step, anchoring urinary molecular signals to spatial cell states to perform non‐invasive atlas‐anchored molecular profiling, is directed at the heterogeneity and the early‐detection gap that defeat current biomarkers. What stands between the concept and the clinic is not a missing technology but a missing program of standardization and prospective validation. Ultimately, the question is empirical and answerable, and the resources needed to answer it are now assembled.

## AUTHOR CONTRIBUTIONS

**Juan Wu**: conceptualization, investigation, visualization, writing—original draft. **Ping Sheng**: investigation, writing—review & editing. **Ying Lu**: investigation, visualization, writing—review & editing. **Ke Chen**: conceptualization, supervision, project administration, writing—review & editing.

## CONFLICT OF INTEREST STATEMENT

The authors declare no conflicts of interest.

## FUNDING INFORMATION

The authors have nothing to report.

## ETHICS STATEMENT

The authors have nothing to report.

## Data Availability

Not applicable. No new data were generated or analysed in this review; all findings discussed are drawn from previously published, publicly available sources cited in the reference list. No primary or public dataset was downloaded, reprocessed or analysed for this article, and all figures are original schematic illustrations created with BioRender rather than plots of underlying data. The reference atlases and public resources discussed in the text are identified by their original publications in the reference list.
